# Undiscovered Bat Hosts of Filoviruses

**DOI:** 10.1371/journal.pntd.0004815

**Published:** 2016-07-14

**Authors:** Barbara A. Han, John Paul Schmidt, Laura W. Alexander, Sarah E. Bowden, David T. S. Hayman, John M. Drake

**Affiliations:** 1 Cary Institute of Ecosystem Studies, Millbrook, New York, United States of America; 2 Odum School of Ecology, University of Georgia, Athens, Georgia, United States of America; 3 Center for the Ecology of Infectious Diseases, University of Georgia, Athens, Georgia, United States of America; 4 University of California, Integrative Biology, Berkeley, California, United States of America; 5 Molecular Epidemiology and Public Health Laboratory, Hopkirk Research Institute, Massey University, Palmerston North, New Zealand; Armed Forces Health Surveillance Center, UNITED STATES

## Abstract

Ebola and other filoviruses pose significant public health and conservation threats by causing high mortality in primates, including humans. Preventing future outbreaks of ebolavirus depends on identifying wildlife reservoirs, but extraordinarily high biodiversity of potential hosts in temporally dynamic environments of equatorial Africa contributes to sporadic, unpredictable outbreaks that have hampered efforts to identify wild reservoirs for nearly 40 years. Using a machine learning algorithm, generalized boosted regression, we characterize potential filovirus-positive bat species with estimated 87% accuracy. Our model produces two specific outputs with immediate utility for guiding filovirus surveillance in the wild. First, we report a profile of intrinsic traits that discriminates hosts from non-hosts, providing a biological caricature of a filovirus-positive bat species. This profile emphasizes traits describing adult and neonate body sizes and rates of reproductive fitness, as well as species’ geographic range overlap with regions of high mammalian diversity. Second, we identify several bat species ranked most likely to be filovirus-positive on the basis of intrinsic trait similarity with known filovirus-positive bats. New bat species predicted to be positive for filoviruses are widely distributed outside of equatorial Africa, with a majority of species overlapping in Southeast Asia. Taken together, these results spotlight several potential host species and geographical regions as high-probability targets for future filovirus surveillance.

## Author Summary

Preventing future outbreaks of ebolaviruses in humans and other vulnerable animal populations will require identifying the natural reservoirs of filoviruses. Accumulating indirect evidence points to certain bat species as prime suspects. To guide the search for natural filovirus reservoirs, we mined intrinsic biological data on the world’s bat species to determine what features best predict filovirus hosts compared to bats at large. We report a suite of traits that distinguishes seropositive bat species from all others with an estimated 87% accuracy. We also identify several bat species not currently known to be filovirus hosts whose trait profiles indicate should be surveillance targets. Geographic regions where numerous potential filovirus hosts co-occur (potential filovirus hotspots) suggest that filovirus distribution and diversity may be greater than previously thought.

## Introduction

After more than 40 years, the natural reservoirs of viruses in the genus Ebolavirus remain elusive. Accumulating indirect evidence during this time points to bats as primary suspects because several species have been found positive for filovirus antibodies (S1 Table). Some of these species have also been confirmed as natural reservoirs for another filovirus, Marburg virus [1,2]. Three bat species demonstrate the ability to replicate ebolavirus following experimental inoculation [3], and ebolavirus RNA has been discovered in three, naturally infected species [4]. In contrast to other surveyed mammal species (great apes, duiker), there is little evidence of filovirus-induced morbidity in bats [1]. Such asymptomatic infections make bats more likely to be natural reservoir candidates for ebolaviruses than, for example, great apes (gorilla and chimpanzee), which suffer mortality rates exceeding those observed in human populations [5].

Effective surveillance in the countries most frequently affected by ebolaviruses (e.g., Uganda, Democratic Republic of Congo [6], and the countries affected by the recent outbreak in West Africa [7]) is hampered by the incredible diversity of species over such a large geographical area. For example, West Africa is recognized as one of the most species-rich regions on Earth, with a large number of endemic species typically present in low densities [8,9]. Moreover, there is pronounced seasonality in regions affected by ebolaviruses with wet and dry seasons contributing to fruiting phenology and water availability that combine to influence the movement ecology, breeding, and birth pulses in a number of species, including bats [10–12]. Though wildlife surveillance to date surpasses 30,000 individuals collected from hundreds of species, we have yet to isolate live ebolavirus from any African wild species.

What other species might be natural hosts of filoviruses in the wild? To answer this question, we applied a machine learning approach to mine patterns in data on the world’s bat species. Here, we report an intrinsic trait profile that distinguishes seropositive bat species from all others with an estimated 87% accuracy. We identify a rank order of particular bat species whose trait profiles suggest a high probability that they could also be permissive to filovirus infection, and geographic regions where numerous of these potentially novel filovirus hosts co-occur to highlight surveillance targets of candidate reservoir species.

## Materials and Methods

For all 1116 bat species, we collected life history, physiological and ecological traits from PanTHERIA [13], a species-level database of the world’s mammals (S2 Table). We calculated 3 additional, derivative traits from basic morphological and demographic variables: post-natal growth rate (weaning body mass/neonatal body mass); relative age to sexual maturity (sexual maturity age/maximum longevity); relative age at first birth (age at first birth/maximum longevity). We added bat family as a series of 18 binary variables to explore the likelihood of taxonomic clustering among carriers. We calculated species density, defined as the richness of mammal species found within a species’ geographic range (as reported in IUCN [14]) divided by the total geographic range area for each bat species (*n*/km^2^). We compiled published data on diet and activity patterns [15]; torpor and migratory behavior [16]; and mass-corrected production (the mean mass of offspring produced per year, normalized by adult body size [15]. Bat species names were standardized using Wilson and Reeder 2005 [17].

Each bat species was assigned a binary code according to its current status (0 –not currently known to carry a filovirus; 1 –published evidence; S1 Table). For this binary response variable, we applied generalized boosted regression [18–20], a type of machine learning that seeks to maximize classification accuracy (in this case, discriminating reservoir status among 1116 bat species) by learning the patterns of features that distinguish between bats that have tested positive for filoviruses from all other species. Machine learning is particularly well-suited to comparative studies because it does not assume an underlying data distribution [21], and explanatory power is unaffected by collinearity, hidden interactions, and non-random patterns of missing data common in ecological data sets (e.g., those that arise through sampling bias, or when species share similar trait values as a result of phylogenetic relatedness) [22,23]. The model-free approach of machine learning algorithms like generalized boosted regression trees enables superior predictive accuracy based on patterns inherent in data themselves rather than based on *a priori* assumptions about underlying ecological processes or simple parametric relationships, in proportion to the quantity of information contained in the data.

Boosted regression trees generate a series of recursive binary splits for randomly sampled predictor variables. Each successive tree is built using the residuals of the previous best-performing tree as the new response variable. Thus, an ensemble of linked trees is generated where each tree achieves increasingly more accurate classification based on randomly selected variables. In our analyses, we repeated the tree building process several thousand times to create an ensemble classification model of up to 5000 trees. Datasets were partitioned into training (80% of all 1116 species) and test sets (the remaining 20%) prior to analysis. We applied 10-fold cross-validation during model building to prevent over-fitting, and permutation procedures to generate relative importance scores for each predictor variable (S3 Table, which also summarizes tuning parameters, performance metrics (AUC), and complete trait profiles). To calibrate performance, we conducted randomized bootstrapped permutation analysis of the species labels (500 permutations), a procedure referred to as *target shuffling* in business analytics. We calculated a baseline mean AUC for these permutations (0.6) and corrected our test AUC (originally AUC = 0.97) by this baseline to arrive at our corrected test AUC of 0.87 = 0.97-(0.60–0.5). To investigate the sensitivity of our results to errors and permutations in the covariates, we randomly removed 1%, 5%, 10%, 15%, and 20% of trait values, refit the model, and calculated the Spearman rank-order correlation between scores obtained using the corrupted data and those of our original analysis. This exercise showed the algorithm to be extremely adept at identifying the relative risk among bat species (rank order) with up to 5% of data removed (ρ = 0.99) and very good with up to 20% of data removed (ρ = 0.90) (S4 Table). In our analysis, “unknown” carriers (1095 species) were designated “non-carriers”, labeled as 0. In the absence of repeated experimental inoculations, a large number of individuals of each species must be sampled before consensus can be reached that a given species is unable to harbor infection. Thus, we adopted this more conservative designation–essentially presence vs. background–to align with our aim of developing models whose baseline classification performance will continue to improve with future discoveries of new filovirus-positive species.

Intrinsic features that reflect life history and biology are less susceptible to sampling biases than epidemiological data–for example, public heath and research expenditures are unlikely to influence a species’ age to sexual maturity, or other similar life history features. However, to control for any potential effect of sampling bias on our results, we tallied the number of primary literature citations in the Web of Science (WOS) for each bat species in our dataset as a proxy for study effort. Citation count was within the top dozen variables important for predicting filovirus-positive status, but it had low relative importance for prediction accuracy (S2 Table). Removing WOS hits from the analysis did not alter the rank order of variables most important for predicting filovirus-positive bats, confirming that while some filovirus-positive bat species may be better studied than others, studied-ness did not bias the trait profiles generated by our modeling approach. Analyses were performed using the gbm package [19] in R [24].

To identify hot spots of filovirus carriers, we mapped the geographic ranges of all known filovirus-positive bat species (S1 Table), as well as new filovirus carriers in the 90^th^ percentile of model predictions (S3 Table). We also provide maps for species comprising the 95^th^ and 99^th^ percentiles (S1 Fig). All geographic ranges were obtained from the IUCN database of terrestrial mammals [14] and compiled in ArcGIS [25].

## Results and Discussion

From peer-reviewed primary literature, we identified 21 out of 1116 (~1.9%) total extant bat species to have tested positive for any filovirus by means of any diagnostic (i.e., either serological or molecular assays). Approximately half of these species (*n* = 11) are fruit bats belonging to Family Pteropodidae (the Old World fruit bats), and the other half are primarily insectivorous bats from 4 families (S1 Table). Although fruit bats comprise only about 16% of global bat biodiversity (186/1116 extant species), we estimate 5 times as many fruit bat individuals have been sampled for filoviruses compared to insectivorous bats (S2 Table), which corroborates on a global scale the surveillance bias recently reported for ebolaviruses in bats of Africa [26].

**Fig 1:**
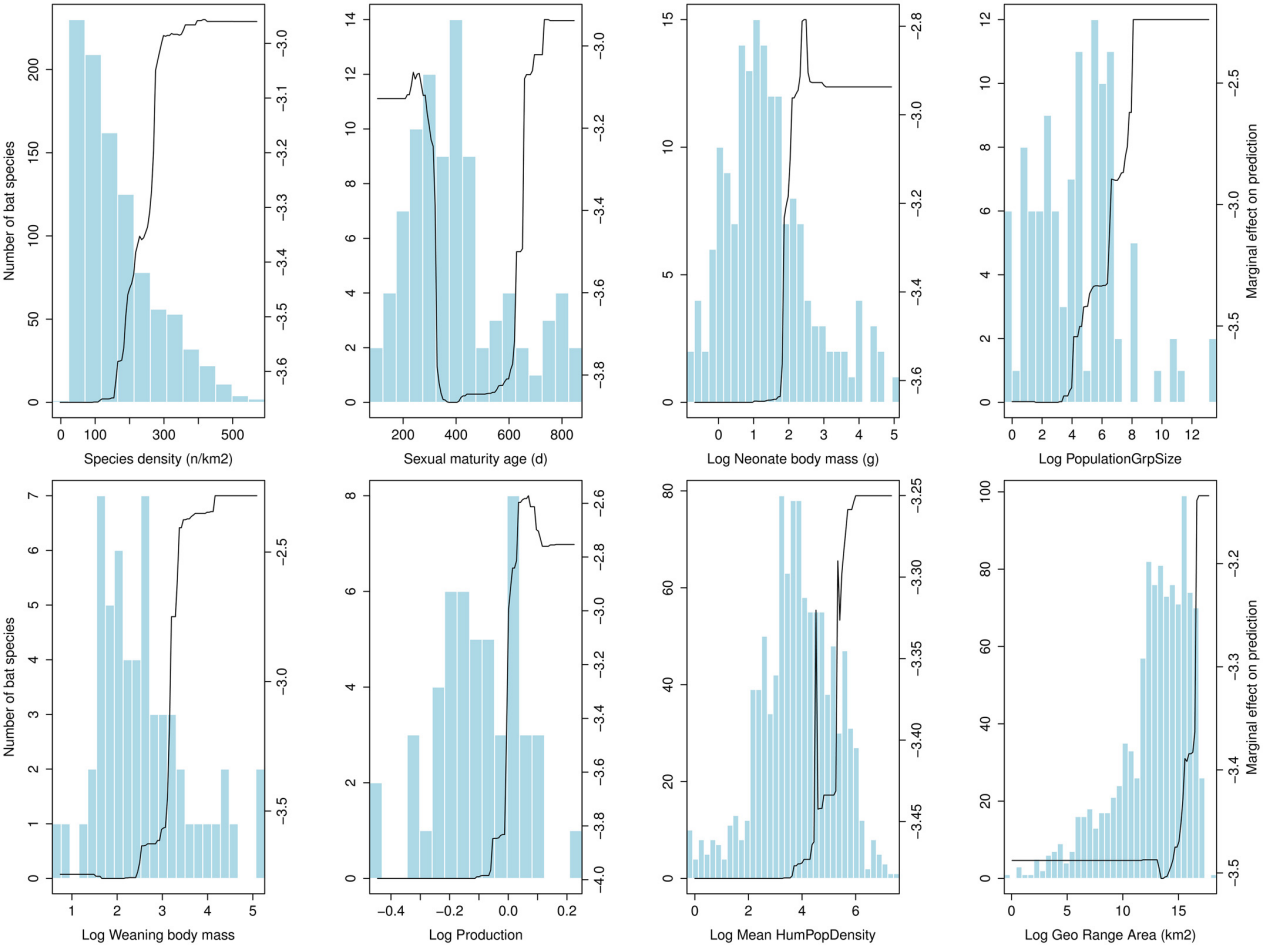
The trait profile of filovirus-positive bat species. Partial dependence plots of the top 8 predictor variables from a generalized boosted regression analysis illustrate the trait profile of bat species that are positive for filoviruses in the wild. Plots appear in order of predictive importance from left to right, top to bottom. Line graphs depict the marginal effect of a given variable for correctly predicting filovirus-positive status in bats. Blue frequency histograms show the distribution of available trait values across all 1116 bat species while the solid curve shows the trait tendencies for filovirus-positive bat species.

Using 57 variables describing the biology, life history, ecology, taxonomy, and biogeography of all bat species (S2 Table), our model predicted filovirus-positivity with 87% accuracy, and revealed a trait profile that distinguishes filovirus-positive species from other bats (Fig 1, S5 Table). In general, filovirus-positive bat species tend to have neonates that are larger at birth and wean at a larger size compared to other bats. This tendency to produce larger offspring was not an artifact of large adult body size. Rather, filovirus-positive bats produce greater biomass for their body size compared to other bat species (the *production* variable [27], Fig 1). The majority of bats have 1 litter per year with a single pup in each litter, but some populations support a second litter in some years (notably among the Vespertilionidae, the most speciose Family of insectivorous bats, and the Pteropodidae, the Old World fruit bats). We found that filovirus-positive species disproportionately display this tendency to have more than a single litter (pup) per year [28]. We also observed a bimodal pattern in sexual maturity age for filovirus-positive species, a pattern we conjecture may arise from small species (insectivorous bats) displaying earlier ages of sexual maturity compared to the large species (fruit bats) in the tropics where reproductive rates of non-hibernating bats decrease with body size [29]. Filovirus-positive species also display a tendency to live in larger population groups (roosts) compared to other bats. While group-living affords many benefits, costs of group living include increased pathogen transmission [30] and conspicuousness to predators, including human hunters [31]. Thus, it is possible that species living in large, conspicuous roosts are displaying compensatory effects of faster reproductive rates (earlier age to sexual maturity [32] or more offspring per year [29]) in response to increased extrinsic mortality risks conferred by hunting pressure. Overall, our results suggest that even though bats are constrained to a relatively slow life history strategy (i.e., long-lived with few offspring per year) compared to similarly sized mammals, filovirus-positive bat species are those whose life history pace is at the leading edge of these constraints. In addition to traits that may enable bats to be more permissive to filovirus infection at the cellular level [33,34], a life history profile reflecting faster reproductive rates may increase the likelihood of infection persistence through the more rapid replenishment of susceptible young [1,28].

**Fig 2:**
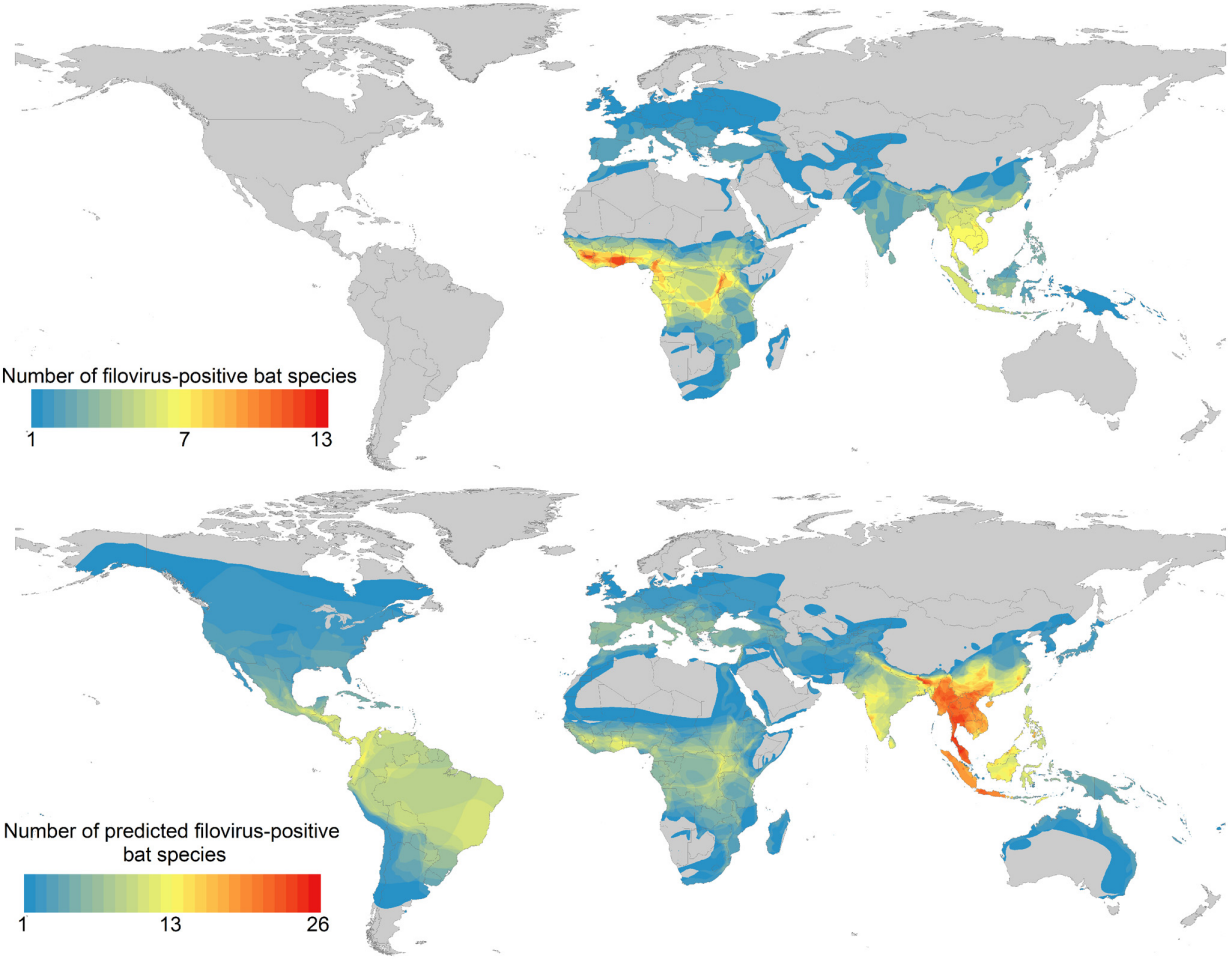
Range maps of known and predicted additional filovirus-positive bat species. Overlapping geographic ranges of 21 bat species that have tested positive for filoviruses (top), and additional bat species predicted to carry filoviruses in the 90^th^ percentile probability through generalized boosted regression analysis (bottom).

**Fig 3:**
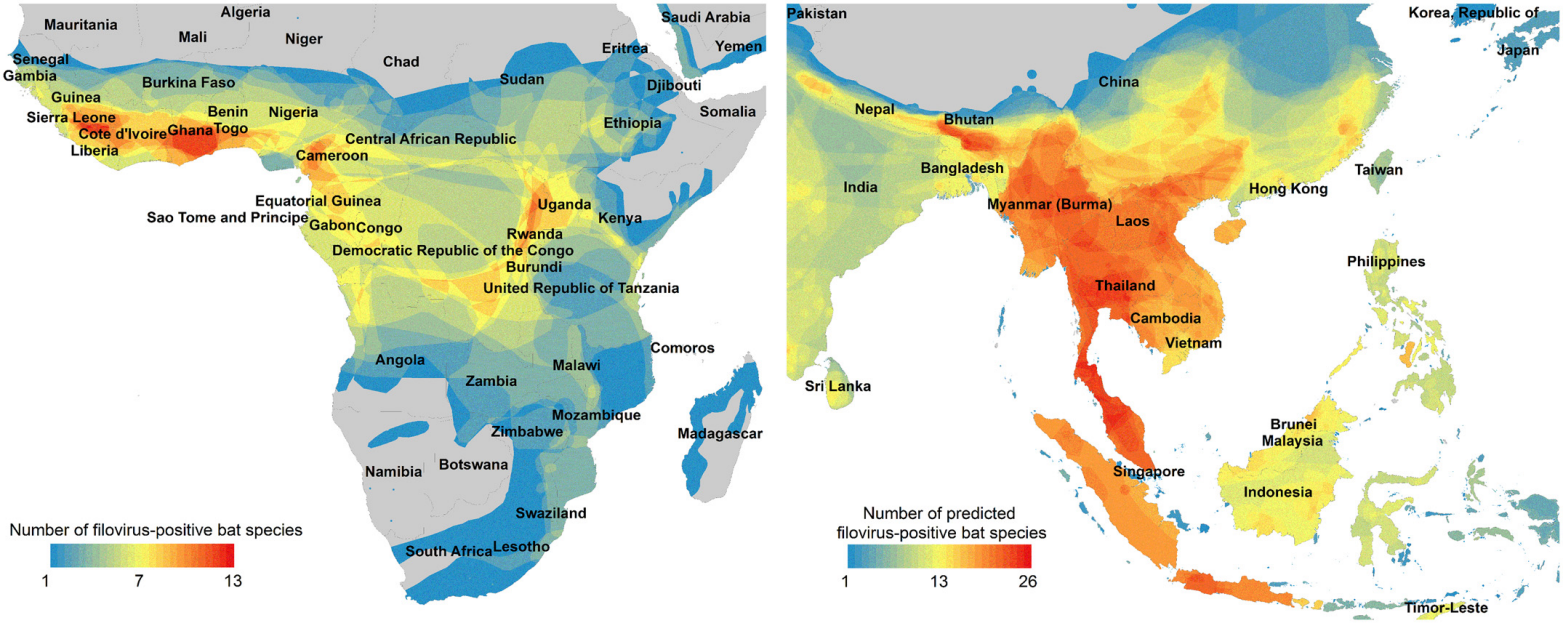
Magnified range maps of known and predicted filovirus-positive bat species. Magnifications of hotspots of filovirus-positive bat species in sub-Saharan Africa (left), and hotspots in Southeast Asia showing overlapping geographic ranges for predicted new filovirus carriers within the 90^th^ percentile probability (right).

Beyond intrinsic fitness components, our analyses revealed that filovirus-positive species exhibit larger geographic ranges containing higher mammal species richness per square kilometer than other bats (species density, Fig 1). Even after correcting for geographic range size, filovirus-positive bat species overlap with a greater diversity of mammal species per square kilometer, a finding that recapitulates a scientific consensus that there are likely to be multiple natural reservoirs supporting filoviruses such as *Zaire ebolavirus* [35]. This result corroborates independent studies within Africa predicting the environmental niche of ebolaviruses to span primary tropical rainforest (continuous tropical rainforests as well as gallery rainforests, which occur along riparian and transitional zones) [36–38]. But, in a departure from previous studies, our analysis identified several hotspots outside Africa where up to 25 predicted filovirus host species overlap in geographic range (Figs 2 and 3; S1 Fig).

Geographic ranges of filovirus-positive bat species are concentrated in sub-Saharan Africa and Southeast Asia, spanning a total of 133 countries (Fig 2a). There is a conspicuous lack of surveillance in the western hemisphere, and to our knowledge there are no published studies reporting the results (positive or negative) of filovirus surveillance efforts in North, Central, or South America [26]. Novel bat carriers predicted by our model (i.e., those in the top 10%) are much more widely distributed than expected, with predicted species occurring across Southeast Asia, and Central and South America (Fig 2b; S3 Table). The predictions in the Americas are intriguing because, while New World bats may exhibit the appropriate traits, biogeographical processes may prevent filoviruses from existing in these regions. Indeed, homologous copies of VP35-like and NP-like gene integrations were found in both Old World and New World species of *Myotis* bats [39]. If filoviruses are discovered in bat species in the Americas, this would call into question the age of the *Filoviridae*, which, through whole genome analyses, have been estimated to share common ancestry 10,000 years ago [40]. Analyses of integrated elements in mammalian genomes, however, suggest filoviruses may be much older [41]. Among the 112 species comprising the 90^th^ percentile probability there are 9 *Myotis* species (S3 Table). Among these, *Myotis ricketti* tested seropositive and *Myotis fimbriatus* tested seronegative for Reston ebolavirus in China [42]. Diagnostic tests of the remaining 7 species have, to our knowledge, never been reported at the species level.

A majority of the newly predicted filovirus carriers overlap in Southeast Asia (Fig 3), with notable hotspots occurring in regions of Thailand, Burma, Malaysia, Vietnam, and northeast India. A recent study reports the negative results of a large survey testing 500 individuals of *Pteropus lylei* for ebolavirus across 10 roosting sites in Thailand. This study was designed with enough statistical power to detect ebolavirus prevalence as low as 6% [43]. Our model ranked this particular fruit bat species behind 195 other bat species in its probability of filovirus-seropositivity. In particular, it is preceded by three other species commonly found in Thailand–*Pipistrellus tenuis*, *Eonycteris spelaea*, and *Megaderma lyra*, which rank 3^rd^, 5^th^, and 7^th^ in a global list of unsurveyed bats predicted to be seropositive (S3 Table). Future surveillance efforts may be streamlined by prioritizing filovirus-testing by species displaying the strongest trait similarities with known filovirus-positive species.

Despite numerous suitable bat hosts and ongoing discoveries of novel filoviruses in this region (e.g., [44]), there are comparatively few reports of disease outbreaks in Asia. Though pigs were identified as possible reservoirs of *Reston ebolavirus* through routine investigation of syndromic disease, there have been no reports of human disease in this region. One outstanding question for future work is to investigate why there are so few spillover events reported for human and wildlife populations in Southeast Asia compared to equatorial Africa. Whether outbreaks are indeed occurring but on a smaller or less easily detectable scale (e.g., as in Ethiopia [45]), or whether filovirus strains in this region are fundamentally less virulent to their host species, sorting the competing hypotheses about why filovirus infection dynamics in Africa differ from those in Asia will begin with more targeted surveillance of candidate reservoir species.

## Supporting Information

S1 Fig Range maps of predicted additional filovirus-positive bat species in the 90^th^, 95^th^, and 99^th^ percentile probability. (PNG)

S1 Table A table containing all published records of bat individuals reported to the species level that have been sampled for filoviruses using a variety of diagnostic methods. From the primary literature (*Reference*), we report the total number of bats tested via PCR (*PCR tested*), antibody tests (*Ab tested*), and the number of virus isolation attempts (*iso attempted indiv*), and the number of bats that tested positive using any of these methods. We also report the specific viral strain or primer that used for diagnostics (*virus*, *primer*), and whether the bat species belongs to Family Pteropodidae (the Old World fruit bats). Figshare DOI: 10.6084/m9.figshare.3114310
(PDF)

S2 Table A list of the coverage and the definitions for all variables included in the boosted regression tree (excluding taxonomic families). Coverage is calculated as a percentage of the total bat species (out of N = 1116) and a percentage of the total filovirus-positive bat species (out of N = 21) for which there were data available for a given variable.
(PDF)

S3 Table Species predictions generated by the generalized boosted regression model. The first 112 species comprise the 90^th^ percentile probability of novel filovirus-positive bat species. Label is a binomial variable denoting filovirus-positivity. Probability is a transformation of model outputs.
(PDF)

S4 Table Spearman’s rank correlation analyses of bat species rankings produced by boosted regression models on complete data compared to data missing 1%-20% of randomly selected traits (covariates). (PDF)

S5 Table Tuning parameters (shrinkage, interaction depth), performance metrics (AUC), and complete trait profiles produced by generalized boosted regression models examining filovirus-positive status as a binary variable (Bernoulli error distribution). The final model includes the number of citations per bat species in Web of Science (WOS_HITS), showing that while study effort is within the top dozen most important predictors of filovirus-positive status, it is less important than intrinsic traits. Variables taken from PanTHERIA retain their original names. We also report here the baseline AUC and standard deviation calculated from a bootstrapped randomized permutation analysis.
(PDF)
