# Supplementary figures and images for: Undiscovered Bat Hosts of Filoviruses
Source: PLoS Negl Trop Dis. 2016 Jul 14;10(7):e0004815. doi: 10.1371/journal.pntd.0004815 (PMC4945033; doi:10.1371/journal.pntd.0004815)

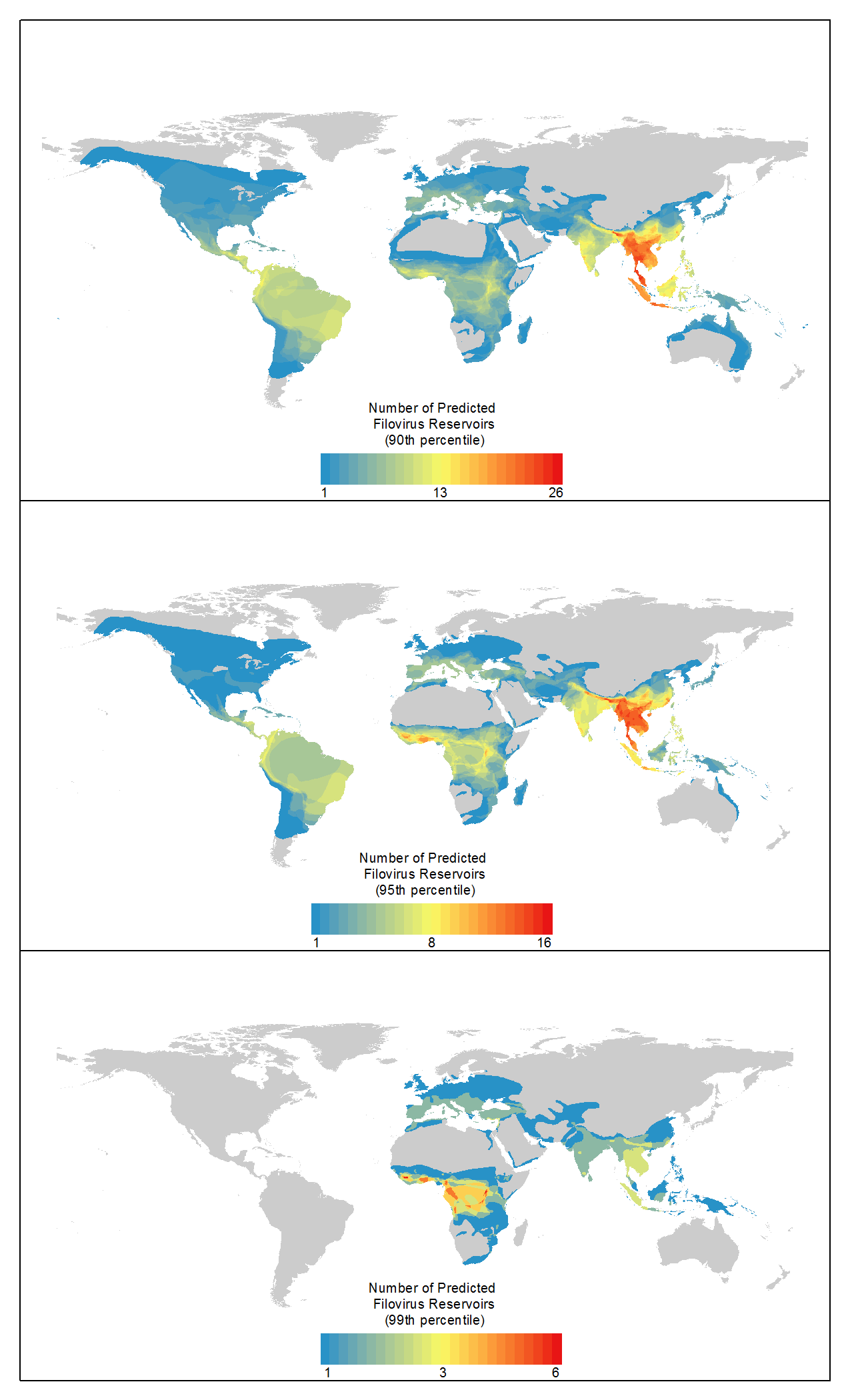

Supplement: S1 Fig — (PNG) [file pntd.0004815.s001.png]
