# Supplementary material for: Undiscovered Bat Hosts of Filoviruses
Source: PLoS Negl Trop Dis. 2016 Jul 14;10(7):e0004815. doi: 10.1371/journal.pntd.0004815 (PMC4945033; doi:10.1371/journal.pntd.0004815)
Supplement: S1 Table — From the primary literature (Reference), we report the total number of bats tested via PCR (PCR tested), antibody tests (Ab tested), and the number of virus isolation attempts (iso attempted indiv), and the number of bats that tested positive using any of these methods. We also report the specific viral strain or primer that used for diagnostics (virus, primer), and whether the bat species belongs to Family Pteropodidae (the Old World fruit bats). Figshare DOI: 10.6084/m9.figshare.3114310 (PDF) [file pntd.0004815.s002.pdf]

Figshare DOI: 10.6084/m9.figshare.3114310

virus	bat_species	Pteropid fruit bat	Ab+	Ab tested	PCR+	PCR tested	primer	min_individuals_tested	isolated+	iso attempted indiv	Reference	Notes
MARV	Rousettus_aegyptiacus	1	250	1622	40	1622	MARV-VP35	1622	7	1622	Amman et al. 2012	
ZEBOV	Chaerephon_major	0	0	26	NA	NA	NA	26	0	26	Breman et al. 1999	
ZEBOV	Eidolon_helvum	1	0	6	NA	NA	NA	6	0	6	Breman et al. 1999	
ZEBOV	Epomops_franqueti	1	0	21	NA	NA	NA	21	0	21	Breman et al. 1999	
ZEBOV	Hipposideros_cyclops	0	0	52	NA	NA	NA	52	0	52	Breman et al. 1999	
ZEBOV	Hipposideros_ruber	0	0	17	NA	NA	NA	17	0	17	Breman et al. 1999	
ZEBOV	Mops_condylurus	0	0	54	NA	NA	NA	54	0	54	Breman et al. 1999	
ZEBOV	Mops_congicus	0	0	20	NA	NA	NA	20	0	20	Breman et al. 1999	
ZEBOV	Mops_nanulus	0	0	15	NA	NA	NA	15	0	15	Breman et al. 1999	
ZEBOV	Mops_thersites	0	0	69	NA	NA	NA	69	0	69	Breman et al. 1999	
ZEBOV	Myotis_bocagii	0	0	17	NA	NA	NA	17	0	17	Breman et al. 1999	
ZEBOV	Neoromicia_nanus	0	0	73	NA	NA	NA	73	0	73	Breman et al. 1999	
ZEBOV	Saccolaimus_peli	0	0	9	NA	NA	NA	9	0	9	Breman et al. 1999	
ZEBOV	Eidolon_helvum	1	1	262	NA	NA	NA	262	NA	NA	Hayman et al. 2010	
MARV	Eidolon_helvum	1	0	262	NA	NA	NA	262	NA	NA	Hayman et al. 2010	
ZEBOV	Hypsignathus_monstrosus	1	0	3	NA	NA	NA	3	NA	NA	Hayman et al. 2010	
MARV	Hypsignathus_monstrosus	1	0	3	NA	NA	NA	3	NA	NA	Hayman et al. 2010	
ZEBOV	Epomophorus_gambianus	1	5	37	NA	NA	NA	37	NA	NA	Hayman et al. 2012a	
RESTV	Epomophorus_gambianus	1	3	37	NA	NA	NA	37	NA	NA	Hayman et al. 2012a	
screen	Epomophorus_gambianus	1	7	37	NA	NA	NA	37	NA	NA	Hayman et al. 2012a	Number that reacted to 1:1 RESTV:ZEBOV screen but not to singly
ZEBOV	Epomops_buettikoferi	1	0	1	NA	NA	NA	1	NA	NA	Hayman et al. 2012a	
RESTV	Epomops_buettikoferi	1	0	1	NA	NA	NA	1	NA	NA	Hayman et al. 2012a	
screen	Epomops_buettikoferi	1	0	1	NA	NA	NA	1	NA	NA	Hayman et al. 2012a	Number that reacted to 1:1 RESTV:ZEBOV screen but not to singly
ZEBOV	Epomops_franqueti	1	3	27	NA	NA	NA	27	NA	NA	Hayman et al. 2012a	
RESTV	Epomops_franqueti	1	0	27	NA	NA	NA	27	NA	NA	Hayman et al. 2012a	
screen	Epomops_franqueti	1	7	27	NA	NA	NA	27	NA	NA	Hayman et al. 2012a	Number that reacted to 1:1 RESTV:ZEBOV screen but not to singly
ZEBOV	Hypsignathus_monstrosus	1	2	16	NA	NA	NA	16	NA	NA	Hayman et al. 2012a	
RESTV	Hypsignathus_monstrosus	1	1	16	NA	NA	NA	16	NA	NA	Hayman et al. 2012a	
screen	Hypsignathus_monstrosus	1	4	16	NA	NA	NA	16	NA	NA	Hayman et al. 2012a	Number that reacted to 1:1 RESTV:ZEBOV screen but not to singly
ZEBOV	Nanonycteris_veldkampii	1	0	4	NA	NA	NA	4	NA	NA	Hayman et al. 2012a	
RESTV	Nanonycteris_veldkampii	1	0	4	NA	NA	NA	4	NA	NA	Hayman et al. 2012a	
screen	Nanonycteris_veldkampii	1	1	4	NA	NA	NA	4	NA	NA	Hayman et al. 2012a	Number that reacted to 1:1 RESTV:ZEBOV screen but not to singly
ZEBOV	Hypsignathus_monstrosus	1	0	1	NA	NA	NA	1	0	1	Germain 1978	Bats may not have been Ab tested- isolation explicitly mentioned, Ab testing suggested by context.
ZEBOV	Casinycteris_argynnis	1	0	1	NA	NA	NA	2	0	2	Leirs et al. 1999	
ZEBOV	Chaerephon_ansorgei	0	0	120	NA	NA	NA	121	0	121	Leirs et al. 1999	
ZEBOV	Chaerephon_pumilus	0	0	210	NA	NA	NA	211	0	211	Leirs et al. 1999	
ZEBOV	Epomops_franqueti	1	0	2	NA	NA	NA	2	0	2	Leirs et al. 1999	
ZEBOV	Megaloglossus_woermanni	1	0	38	NA	NA	NA	43	0	43	Leirs et al. 1999	
ZEBOV	Micropteropus_pusillus	1	0	78	NA	NA	NA	78	0	78	Leirs et al. 1999	
ZEBOV	Miniopterus_minor	0	0	2	NA	NA	NA	2	0	2	Leirs et al. 1999	
ZEBOV	Mops_condylurus	0	0	10	NA	NA	NA	10	0	10	Leirs et al. 1999	
ZEBOV	Mops_nanulus	0	0	14	NA	NA	NA	14	0	14	Leirs et al. 1999	
ZEBOV	Mops_niveiventer	0	0	3	NA	NA	NA	3	0	3	Leirs et al. 1999	
ZEBOV	Mops_thersites	0	0	1	NA	NA	NA	1	0	1	Leirs et al. 1999	
ZEBOV	Myopterus_whitleyi	0	0	1	NA	NA	NA	2	0	2	Leirs et al. 1999	
ZEBOV	Myotis_bocagii	0	0	22	NA	NA	NA	22	0	22	Leirs et al. 1999	
ZEBOV	Neoromicia_malagasyensis	0	0	0	NA	NA	NA	1	0	1	Leirs et al. 1999	No individuals Ab tested (1 isolation attempt)
ZEBOV	Neoromicia_nanus	0	0	2	NA	NA	NA	2	0	2	Leirs et al. 1999	
ZEBOV	Neoromicia_tenuipinnis	0	0	1	NA	NA	NA	1	0	1	Leirs et al. 1999	
ZEBOV	Nycteris_hispida	0	0	1	NA	NA	NA	2	0	2	Leirs et al. 1999	
ZEBOV	Scotophilus_dinganii	0	0	19	NA	NA	NA	20	0	20	Leirs et al. 1999	
ZEBOV	Epomops_franqueti	1	8	117	5	117	FILO-L	117	see note	see note	Leroy et al. 2005	Isolation attempted, all negative, but listed by location not species. Some animals tested for Ab and not PCR and vice-versa. No overlap in PCR/Ab positives, so **number of bats positive is Ab+ plus PCR. Several filo primers used in addition to universal.**
ZEBOV	Hypsignathus_monstrosus	1	4	17	4	21	FILO-L	21	see note	see note	Leroy et al. 2005	Isolation attempted, all negative, but listed by location not species. Some animals tested for Ab and not PCR and vice-versa. No overlap in PCR/Ab positives, so **number of bats positive is Ab+ plus PCR. Several filo primers used in addition to universal.**
ZEBOV	Myonycteris_torquata	1	4	58	4	141	FILO-L	141	see note	see note	Leroy et al. 2005	Isolation attempted, all negative, but listed by location not species. Some animals tested for Ab and not PCR and vice-versa. No overlap in PCR/Ab positives, so **number of bats positive is Ab+ plus PCR. Several filo primers used in addition to universal.**
ZEBOV	Macroglossus_sobrinus	1	0	1	0	1	FILO-L	1	NA	NA	Olival et al. 2013	
RESTV	Macroglossus_sobrinus	1	0	1	0	1	FILO-L	1	NA	NA	Olival et al. 2013	
screen	Macroglossus_sobrinus	1	0	1	0	1	FILO-L	1	NA	NA	Olival et al. 2013	Number that reacted to 1:1 RESTV:ZEBOV screen but not to singly
ZEBOV	Megaderma_lyra	0	0	56	0	56	FILO-L	56	NA	NA	Olival et al. 2013	
RESTV	Megaderma_lyra	0	0	56	0	56	FILO-L	56	NA	NA	Olival et al. 2013	
screen	Megaderma_lyra	0	4	56	0	56	FILO-L	56	NA	NA	Olival et al. 2013	Number that reacted to 1:1 RESTV:ZEBOV screen but not to singly
RESTV	Rousettus_leschenaultii	1	1	141	0	141	FILO-L	141	NA	NA	Olival et al. 2013	One sample more reactive to RESTV than ZEBOV
ZEBOV	Rousettus_leschenaultii	1	4	141	0	141	FILO-L	141	NA	NA	Olival et al. 2013	
screen	Rousettus_leschenaultii	1	15	141	0	141	FILO-L	141	NA	NA	Olival et al. 2013	Number that reacted to 1:1 RESTV:ZEBOV screen but not to singly
ZEBOV	Epomops_franqueti	1	25	725	NA	NA	NA	725	NA	NA	Pourrut et al. 2007	
ZEBOV	Hypsignathus_monstrosus	1	5	111	NA	NA	NA	111	NA	NA	Pourrut et al. 2007	
ZEBOV	Myonycteris_torquata	1	10	554	NA	NA	NA	554	NA	NA	Pourrut et al. 2007	
ZEBOV	Casinycteris_argynnis	1	0	18	NA	NA	NA	18	NA	NA	Pourrut et al. 2009	
MARV	Casinycteris_argynnis	1	0	18	NA	NA	NA	18	NA	NA	Pourrut et al. 2009	
ZEBOV	Eidolon_helvum	1	0	49	NA	NA	NA	49	NA	NA	Pourrut et al. 2009	
MARV	Eidolon_helvum	1	0	47	NA	NA	NA	47	NA	NA	Pourrut et al. 2009	
MARV	Epomops_franqueti	1	2	679	NA	NA	NA	679	NA	NA	Pourrut et al. 2009	
ZEBOV	Epomops_franqueti	1	36	805	NA	NA	NA	805	NA	NA	Pourrut et al. 2009	
ZEBOV	Hipposideros_gigas	0	3	24	NA	NA	NA	24	NA	NA	Pourrut et al. 2009	H. gigas samples were pooled with M. condylurus samples in this study; positive results are for the pooled sample containing both species.
MARV	Hypsignathus_monstrosus	1	1	103	NA	NA	NA	103	NA	NA	Pourrut et al. 2009	
ZEBOV	Hypsignathus_monstrosus	1	9	125	NA	NA	NA	125	NA	NA	Pourrut et al. 2009	
MARV	Megaloglossus_woermanni	1	0	39	NA	NA	NA	39	NA	NA	Pourrut et al. 2009	
ZEBOV	Megaloglossus_woermanni	1	0	49	NA	NA	NA	49	NA	NA	Pourrut et al. 2009	
MARV	Micropteropus_pusillus	1	1	177	NA	NA	NA	177	NA	NA	Pourrut et al. 2009	
ZEBOV	Micropteropus_pusillus	1	4	197	NA	NA	NA	197	NA	NA	Pourrut et al. 2009	
ZEBOV	Mops_condylurus	0	3	24	NA	NA	NA	24	NA	NA	Pourrut et al. 2009	H. gigas samples were pooled with M. condylurus samples in this study; positive results are for the pooled sample containing both species.
MARV	Myonycteris_torquata	1	0	493	NA	NA	NA	493	NA	NA	Pourrut et al. 2009	
ZEBOV	Myonycteris_torquata	1	19	573	NA	NA	NA	573	NA	NA	Pourrut et al. 2009	
MARV	Rousettus_aegyptiacus	1	21	299	NA	NA	NA	299	NA	NA	Pourrut et al. 2009	
ZEBOV	Rousettus_aegyptiacus	1	24	307	NA	NA	NA	307	NA	NA	Pourrut et al. 2009	
ZEBOV	Casinycteris_ophiodon	1	0	1	0	1	FILO-L	1	NA	NA	Saéz et al. 2015	"Attempts to demonstrate the presence of Ebola antibodies against Ebola viruses were inconclusive"
ZEBOV	Chaerephon_pumilus	0	0	1	0	1	FILO-L	1	NA	NA	Saéz et al. 2015	"Attempts to demonstrate the presence of Ebola antibodies against Ebola viruses were inconclusive"
ZEBOV	Eidolon_helvum	1	0	6	0	6	FILO-L	6	NA	NA	Saéz et al. 2015	"Attempts to demonstrate the presence of Ebola antibodies against Ebola viruses were inconclusive"
ZEBOV	Epomops_buettikoferi	1	0	17	0	17	FILO-L	17	NA	NA	Saéz et al. 2015	"Attempts to demonstrate the presence of Ebola antibodies against Ebola viruses were inconclusive"
ZEBOV	Hipposideros_caffer	0	0	18	0	18	FILO-L	18	NA	NA	Saéz et al. 2015	"Attempts to demonstrate the presence of Ebola antibodies against Ebola viruses were inconclusive"
ZEBOV	Hipposideros_cyclops	0	0	23	0	23	FILO-L	23	NA	NA	Saéz et al. 2015	"Attempts to demonstrate the presence of Ebola antibodies against Ebola viruses were inconclusive"
ZEBOV	Hipposideros_jonesi	0	0	1	0	1	FILO-L	1	NA	NA	Saéz et al. 2015	"Attempts to demonstrate the presence of Ebola antibodies against Ebola viruses were inconclusive"
ZEBOV	Hypsignathus_monstrosus	1	0	1	0	1	FILO-L	1	NA	NA	Saéz et al. 2015	"Attempts to demonstrate the presence of Ebola antibodies against Ebola viruses were inconclusive"
ZEBOV	Lissonycteris_angolensis	1	0	45	0	45	FILO-L	45	NA	NA	Saéz et al. 2015	"Attempts to demonstrate the presence of Ebola antibodies against Ebola viruses were inconclusive"
ZEBOV	Megaloglossus_woermanni	1	0	3	0	3	FILO-L	3	NA	NA	Saéz et al. 2015	"Attempts to demonstrate the presence of Ebola antibodies against Ebola viruses were inconclusive"
ZEBOV	Mops_condylurus	0	0	1	0	1	FILO-L	1	NA	NA	Saéz et al. 2015	"Attempts to demonstrate the presence of Ebola antibodies against Ebola viruses were inconclusive"
ZEBOV	Myonycteris_leptodon	1	0	21	0	21	FILO-L	21	NA	NA	Saéz et al. 2015	"Attempts to demonstrate the presence of Ebola antibodies against Ebola viruses were inconclusive"
ZEBOV	Nanonycteris_veldkampii	1	0	17	0	17	FILO-L	17	NA	NA	Saéz et al. 2015	"Attempts to demonstrate the presence of Ebola antibodies against Ebola viruses were inconclusive"
MARV	Hipposideros_caffer	0	0	10	0	7	MARV-VP35	13	0	13	Swanepoel et al. 2007	
MARV	Hipposideros_commersoni	0	0	16	0	13	MARV-VP35	17	0	17	Swanepoel et al. 2007	
MARV	Lissonycteris_angolensis	1	0	3	0	3	MARV-VP35	3	0	3	Swanepoel et al. 2007	
MARV	Miniopterus_inflatus	0	**0**	34	1	33	MARV-VP35	38	0	38	Swanepoel et al. 2007	
MARV	Nycteris_hispida	0	0	1	0	1	MARV-VP35	1	0	1	Swanepoel et al. 2007	
MARV	Rhinolophus_eloquens	0	20	206	7	197	MARV-VP35	222	0	222	Swanepoel et al. 2007	
MARV	Rhinolophus_landeri	0	0	0	0	0	MARV-VP35	1	0	1	Swanepoel et al. 2007	No individuals Ab tested (1 isolation attempt)
MARV	Rousettus_aegyptiacus	1	32	156	4	127	MARV-VP35	230	0	230	Swanepoel et al. 2007	
universal	Hipposideros_caffer	0	see note	see note	0	13	FILO-L	13	see note	see note	Swanepoel et al. 2007	Same # of isolation attempts as PCR. More FILO-L sequencing attempts than MARV-specific, but a MARV-specific PCR came back positive (no positives for FILO-L).
universal	Hipposideros_commersoni	0	see note	see note	0	17	FILO-L	17	see note	see note	Swanepoel et al. 2007	Same # of isolation attempts as PCR. More FILO-L sequencing attempts than MARV-specific, but a MARV-specific PCR came back positive (no positives for FILO-L).
universal	Lissonycteris_angolensis	1	see note	see note	0	3	FILO-L	3	see note	see note	Swanepoel et al. 2007	Same # of isolation attempts as PCR. More FILO-L sequencing attempts than MARV-specific, but a MARV-specific PCR came back positive (no positives for FILO-L).
universal	Miniopterus_inflatus	0	see note	see note	0	38	FILO-L	38	see note	see note	Swanepoel et al. 2007	Same # of isolation attempts as PCR. More FILO-L sequencing attempts than MARV-specific, but a MARV-specific PCR came back positive (no positives for FILO-L).
universal	Nycteris_hispida	0	see note	see note	0	1	FILO-L	1	see note	see note	Swanepoel et al. 2007	Same # of isolation attempts as PCR. More FILO-L sequencing attempts than MARV-specific, but a MARV-specific PCR came back positive (no positives for FILO-L).

universal	Rhinolophus_eloquens	0	see note	see note	0	222	FILO-L	222	see note	see note	Swanepoel et al. 2007	Same # of isolation attempts as PCR. More FILO-L sequencing attempts than MARV-specific, but a MARV-specific PCR came back positive (no positives for FILO-L).
universal	Rhinolophus_landeri	0	see note	see note	0	1	FILO-L	1	see note	see note	Swanepoel et al. 2007	Same # of isolation attempts as PCR. More FILO-L sequencing attempts than MARV-specific, but a MARV-specific PCR came back positive (no positives for FILO-L).
universal	Rousettus_aegyptiacus	1	see note	see note	0	230	FILO-L	230	see note	see note	Swanepoel et al. 2007	Same # of isolation attempts as PCR. More FILO-L sequencing attempts than MARV-specific, but a MARV-specific PCR came back positive (no positives for FILO-L).
RESTV	Cynopterus_brachyotis	1	0	35	NA	NA	NA	35	NA	NA	Taniguchi et al. 2011	
RESTV	Emballonura_alecto	0	0	9	NA	NA	NA	9	NA	NA	Taniguchi et al. 2011	
RESTV	Eonycteris_spelaea	1	0	5	NA	NA	NA	5	NA	NA	Taniguchi et al. 2011	
RESTV	Haplonycteris_fischeri	1	0	6	NA	NA	NA	6	NA	NA	Taniguchi et al. 2011	
RESTV	Hipposideros_diadema	0	0	1	NA	NA	NA	1	NA	NA	Taniguchi et al. 2011	
RESTV	Macroglossus_minimus	1	0	2	NA	NA	NA	2	NA	NA	Taniguchi et al. 2011	
RESTV	Miniopterus_australis	0	0	8	NA	NA	NA	8	NA	NA	Taniguchi et al. 2011	
RESTV	Miniopterus_macrotarsus	0	0	1	NA	NA	NA	1	NA	NA	Taniguchi et al. 2011	
RESTV	Miniopterus_schreibersii	0	0	8	NA	NA	NA	8	NA	NA	Taniguchi et al. 2011	
RESTV	Miniopterus_tristis	0	0	1	NA	NA	NA	1	NA	NA	Taniguchi et al. 2011	
RESTV	Pipistrellus_javanicus	0	0	2	NA	NA	NA	2	NA	NA	Taniguchi et al. 2011	
RESTV	Ptenochirus_jagori	1	0	38	NA	NA	NA	38	NA	NA	Taniguchi et al. 2011	
RESTV	Rhinolophus_arcuatus	0	0	1	NA	NA	NA	1	NA	NA	Taniguchi et al. 2011	
RESTV	Rhinolophus_rufus	0	0	2	NA	NA	NA	2	NA	NA	Taniguchi et al. 2011	
RESTV	Rousettus_amplexicaudatus	1	7	16	0	16	RESTV-NP	16	NA	NA	Taniguchi et al. 2011	
RESTV	Scotophilus_kuhlii	0	0	5	NA	NA	NA	5	NA	NA	Taniguchi et al. 2011	
MARV	Casinycteris_argynnis	1	0	0	0	2	MARV-VP35	2	NA	NA	Towner et al. 2007	
MARV	Eidolon_helvum	1	0	33	0	35	MARV-VP35	35	NA	NA	Towner et al. 2007	Ab testing only for bats at sites with PCR+ individuals.
MARV	Epomops_franqueti	1	0	47	0	296	MARV-VP35	296	NA	NA	Towner et al. 2007	Ab testing only for bats at sites with PCR+ individuals.
MARV	Hipposideros_gigas	0	0	1	0	1	MARV-VP35	1	NA	NA	Towner et al. 2007	Ab testing only for bats at sites with PCR+ individuals.
MARV	Hypsignathus_monstrosus	1	0	12	0	56	MARV-VP35	56	NA	NA	Towner et al. 2007	Ab testing only for bats at sites with PCR+ individuals.
MARV	Megaloglossus_woermanni	1	0	20	0	37	MARV-VP35	37	NA	NA	Towner et al. 2007	Ab testing only for bats at sites with PCR+ individuals.
MARV	Micropteropus_pusillus	1	0	19	0	149	MARV-VP35	149	NA	NA	Towner et al. 2007	Ab testing only for bats at sites with PCR+ individuals.
MARV	Myonycteris_torquata	1	0	55	0	264	MARV-VP35	264	NA	NA	Towner et al. 2007	Ab testing only for bats at sites with PCR+ individuals.
MARV	Rousettus_aegyptiacus	1	29	242	4	283	MARV-VP35	283	NA	NA	Towner et al. 2007	Ab testing only for bats at sites with PCR+ individuals.
MARV	Rousettus_aegyptiacus	1	13	546	31	611	MARV-VP35	611	5	see note	Towner et al. 2009	546 Ab tested. 13 definitely positive, 78 intermediate, 455 definite negatives. N and VP40 primers also used. Virus isolated from 5 bats, unclear how many tested.
RESTV	Cynopterus_sphinx	1	2	2	see note	see note	FILO-L	2	NA	NA	Yuan et al. 2012	Screen RESTV only, Ab+ retested with RESTV and ZEBOV (authors recommend 1:1 RESTV:ZEBOV to detect more.) PCR testing was done for some individuals, all came back negative, results not given by species.
RESTV	Hipposideros_armiger	0	0	41	see note	see note	FILO-L	41	NA	NA	Yuan et al. 2012	Screen RESTV only, Ab+ retested with RESTV and ZEBOV (authors recommend 1:1 RESTV:ZEBOV to detect more.) PCR testing was done for some individuals, all came back negative, results not given by species.
RESTV	Hipposideros_cineraceus	0	0	111	see note	see note	FILO-L	111	NA	NA	Yuan et al. 2012	Screen RESTV only, Ab+ retested with RESTV and ZEBOV (authors recommend 1:1 RESTV:ZEBOV to detect more.) PCR testing was done for some individuals, all came back negative, results not given by species.
RESTV	Hipposideros_larvatus	0	0	21	see note	see note	FILO-L	21	NA	NA	Yuan et al. 2012	Screen RESTV only, Ab+ retested with RESTV and ZEBOV (authors recommend 1:1 RESTV:ZEBOV to detect more.) PCR testing was done for some individuals, all came back negative, results not given by species.
RESTV	Hipposideros_pomona	0	3	39	see note	see note	FILO-L	39	NA	NA	Yuan et al. 2012	Screen RESTV only, Ab+ retested with RESTV and ZEBOV (authors recommend 1:1 RESTV:ZEBOV to detect more.) PCR testing was done for some individuals, all came back negative, results not given by species.
RESTV	Miniopterus_schreibersii	0	2	23	see note	see note	FILO-L	23	NA	NA	Yuan et al. 2012	Screen RESTV only, Ab+ retested with RESTV and ZEBOV (authors recommend 1:1 RESTV:ZEBOV to detect more.) PCR testing was done for some individuals, all came back negative, results not given by species.
RESTV	Myotis_chinensis	0	0	6	see note	see note	FILO-L	6	NA	NA	Yuan et al. 2012	Screen RESTV only, Ab+ retested with RESTV and ZEBOV (authors recommend 1:1 RESTV:ZEBOV to detect more.) PCR testing was done for some individuals, all came back negative, results not given by species.
RESTV	Myotis_daubentonii	0	0	24	see note	see note	FILO-L	24	NA	NA	Yuan et al. 2012	Screen RESTV only, Ab+ retested with RESTV and ZEBOV (authors recommend 1:1 RESTV:ZEBOV to detect more.) PCR testing was done for some individuals, all came back negative, results not given by species.
RESTV	Myotis_davidii	0	0	5	see note	see note	FILO-L	5	NA	NA	Yuan et al. 2012	Screen RESTV only, Ab+ retested with RESTV and ZEBOV (authors recommend 1:1 RESTV:ZEBOV to detect more.) PCR testing was done for some individuals, all came back negative, results not given by species.
RESTV	Myotis_fimbriatus	0	0	2	see note	see note	FILO-L	2	NA	NA	Yuan et al. 2012	Screen RESTV only, Ab+ retested with RESTV and ZEBOV (authors recommend 1:1 RESTV:ZEBOV to detect more.) PCR testing was done for some individuals, all came back negative, results not given by species.
RESTV	Myotis_ricketti	0	4	83	see note	see note	FILO-L	83	NA	NA	Yuan et al. 2012	Screen RESTV only, Ab+ retested with RESTV and ZEBOV (authors recommend 1:1 RESTV:ZEBOV to detect more.) PCR testing was done for some individuals, all came back negative, results not given by species.
RESTV	Pipistrellus_pipistrellus	0	4	35	see note	see note	FILO-L	35	NA	NA	Yuan et al. 2012	Screen RESTV only, Ab+ retested with RESTV and ZEBOV (authors recommend 1:1 RESTV:ZEBOV to detect more.) PCR testing was done for some individuals, all came back negative, results not given by species.
RESTV	Rhinolophus_affinis	0	1	69	see note	see note	FILO-L	69	NA	NA	Yuan et al. 2012	Screen RESTV only, Ab+ retested with RESTV and ZEBOV (authors recommend 1:1 RESTV:ZEBOV to detect more.) PCR testing was done for some individuals, all came back negative, results not given by species.
RESTV	Rhinolophus_ferrumequinum	0	0	15	see note	see note	FILO-L	15	NA	NA	Yuan et al. 2012	Screen RESTV only, Ab+ retested with RESTV and ZEBOV (authors recommend 1:1 RESTV:ZEBOV to detect more.) PCR testing was done for some individuals, all came back negative, results not given by species.
RESTV	Rhinolophus_pearsonii	0	0	3	see note	see note	FILO-L	3	NA	NA	Yuan et al. 2012	Screen RESTV only, Ab+ retested with RESTV and ZEBOV (authors recommend 1:1 RESTV:ZEBOV to detect more.) PCR testing was done for some individuals, all came back negative, results not given by species.
RESTV	Rhinolophus_pusillus	0	0	14	see note	see note	FILO-L	14	NA	NA	Yuan et al. 2012	Screen RESTV only, Ab+ retested with RESTV and ZEBOV (authors recommend 1:1 RESTV:ZEBOV to detect more.) PCR testing was done for some individuals, all came back negative, results not given by species.
RESTV	Rhinolophus_sinicus	0	0	6	see note	see note	FILO-L	6	NA	NA	Yuan et al. 2012	Screen RESTV only, Ab+ retested with RESTV and ZEBOV (authors recommend 1:1 RESTV:ZEBOV to detect more.) PCR testing was done for some individuals, all came back negative, results not given by species.
RESTV	Rousettus_leschenaultii	1	11	126	see note	see note	FILO-L	126	NA	NA	Yuan et al. 2012	Screen RESTV only, Ab+ retested with RESTV and ZEBOV (authors recommend 1:1 RESTV:ZEBOV to detect more.) PCR testing was done for some individuals, all came back negative, results not given by species.
RESTV	Scotophilus_kuhlii	0	1	25	see note	see note	FILO-L	25	NA	NA	Yuan et al. 2012	Screen RESTV only, Ab+ retested with RESTV and ZEBOV (authors recommend 1:1 RESTV:ZEBOV to detect more.) PCR testing was done for some individuals, all came back negative, results not given by species.
ZEBOV	Miniopterus_schreibersii	0	1	23	see note	see note	FILO-L	23	NA	NA	Yuan et al. 2012	Screened with RESTV; 32 RESTV+, 25 ZEBOV Ab+ ELISA and/or WB; not all positives listed by species.
ZEBOV	Rousettus_leschenaultii	1	10	126	see note	see note	FILO-L	126	NA	NA	Yuan et al. 2012	Screened with RESTV; 32 RESTV+, 25 ZEBOV Ab+ ELISA and/or WB; not all positives listed by species.
ZEBOV	Cynopterus_sphinx	1	1	2	see note	see note	FILO-L	2	NA	NA	Yuan et al. 2012	Screened with RESTV; 32 RESTV+, 25 ZEBOV Ab+ ELISA and/or WB; not all positives listed by species.
ZEBOV	Hipposideros_pomona	0	1	39	see note	see note	FILO-L	39	NA	NA	Yuan et al. 2012	Screened with RESTV; 32 RESTV+, 25 ZEBOV Ab+ ELISA and/or WB; not all positives listed by species.
ZEBOV	Pipistrellus_pipistrellus	0	2	35	see note	see note	FILO-L	35	NA	NA	Yuan et al. 2012	Screened with RESTV; 32 RESTV+, 25 ZEBOV Ab+ ELISA and/or WB; not all positives listed by species.
ZEBOV	Eidolon_helvum	1	19	748	0	748	FILO-NP	748	NA	NA	Ogawa et al. 2015	Screened for all Ab, universal primers used.
SUDV	Eidolon_helvum	1	19	748	0	748	FILO-NP	748	NA	NA	Ogawa et al. 2015	Screened for all Ab, universal primers used.
RESTV	Eidolon_helvum	1	0	748	0	748	FILO-NP	748	NA	NA	Ogawa et al. 2015	Screened for all Ab, universal primers used.
TAFV	Eidolon_helvum	1	9	748	0	748	FILO-NP	748	NA	NA	Ogawa et al. 2015	Screened for all Ab, universal primers used.
BDBV	Eidolon_helvum	1	8	748	0	748	FILO-NP	748	NA	NA	Ogawa et al. 2015	Screened for all Ab, universal primers used.
MARV	Eidolon_helvum	1	7	748	0	748	FILO-NP	748	NA	NA	Ogawa et al. 2015	Screened for all Ab, universal primers used. Authors tested 748, not 300, for MARV.
ZEBOV	Pteropus_lylei	1	0	500	NA	NA	NA	500	NA	NA	Wacharapluesadee et al. 2015	
universal	Cynopterus_brachyotis	1	NA	NA	0	10	FILO-L	10	NA	NA	Wacharapluesadee et al. 2015	Used consensus primer (FILO-L) for all filoviruses
universal	Cynopterus_sphinx	1	NA	NA	0	4	FILO-L	4	NA	NA	Wacharapluesadee et al. 2015	Used consensus primer (FILO-L) for all filoviruses
universal	Eonycteris_spelaea	1	NA	NA	0	12	FILO-L	12	NA	NA	Wacharapluesadee et al. 2015	Used consensus primer (FILO-L) for all filoviruses
universal	Macroglossus_sobrinus	1	NA	NA	0	2	FILO-L	2	NA	NA	Wacharapluesadee et al. 2015	Used consensus primer (FILO-L) for all filoviruses
universal	Megaerops_niphanae	1	NA	NA	0	1	FILO-L	1	NA	NA	Wacharapluesadee et al. 2015	Used consensus primer (FILO-L) for all filoviruses
universal	Rousettus_amplexicaudatus	1	NA	NA	0	3	FILO-L	3	NA	NA	Wacharapluesadee et al. 2015	Used consensus primer (FILO-L) for all filoviruses
universal	Hipposideros_armiger	0	NA	NA	0	113	FILO-L	113	NA	NA	Wacharapluesadee et al. 2015	Used consensus primer (FILO-L) for all filoviruses
universal	Hipposideros_cineraceus	0	NA	NA	0	4	FILO-L	4	NA	NA	Wacharapluesadee et al. 2015	Used consensus primer (FILO-L) for all filoviruses
universal	Hipposideros_larvatus	0	NA	NA	0	33	FILO-L	33	NA	NA	Wacharapluesadee et al. 2015	Used consensus primer (FILO-L) for all filoviruses
universal	Hipposideros_lekaguli	0	NA	NA	0	158	FILO-L	158	NA	NA	Wacharapluesadee et al. 2015	Used consensus primer (FILO-L) for all filoviruses
universal	Megaderma_lyra	0	NA	NA	0	1	FILO-L	1	NA	NA	Wacharapluesadee et al. 2015	Used consensus primer (FILO-L) for all filoviruses
universal	Miniopterus_magnate	0	NA	NA	0	132	FILO-L	132	NA	NA	Wacharapluesadee et al. 2015	Used consensus primer (FILO-L) for all filoviruses
universal	Miniopterus_pusillus	0	NA	NA	0	1	FILO-L	1	NA	NA	Wacharapluesadee et al. 2015	Used consensus primer (FILO-L) for all filoviruses
universal	Miniopterus_schreibersii	0	NA	NA	0	22	FILO-L	22	NA	NA	Wacharapluesadee et al. 2015	Used consensus primer (FILO-L) for all filoviruses
universal	Myotis_horsfieldi	0	NA	NA	0	6	FILO-L	6	NA	NA	Wacharapluesadee et al. 2015	Used consensus primer (FILO-L) for all filoviruses
universal	Myotis_muricola	0	NA	NA	0	1	FILO-L	1	NA	NA	Wacharapluesadee et al. 2015	Used consensus primer (FILO-L) for all filoviruses
universal	Rhinolophus_shameli	0	NA	NA	0	44	FILO-L	44	NA	NA	Wacharapluesadee et al. 2015	Used consensus primer (FILO-L) for all filoviruses
universal	Rhinolophus_coelophyllus	0	NA	NA	0	7	FILO-L	7	NA	NA	Wacharapluesadee et al. 2015	Used consensus primer (FILO-L) for all filoviruses
universal	Rhinolophus_luctus	0	NA	NA	0	1	FILO-L	1	NA	NA	Wacharapluesadee et al. 2015	Used consensus primer (FILO-L) for all filoviruses
universal	Rhinolophus_malayanus	0	NA	NA	0	4	FILO-L	4	NA	NA	Wacharapluesadee et al. 2015	Used consensus primer (FILO-L) for all filoviruses
universal	Rhinolophus_microglobosus	0	NA	NA	0	1	FILO-L	1	NA	NA	Wacharapluesadee et al. 2015	Used consensus primer (FILO-L) for all filoviruses
universal	Rhinolophus_pusillus	0	NA	NA	0	1	FILO-L	1	NA	NA	Wacharapluesadee et al. 2015	Used consensus primer (FILO-L) for all filoviruses
universal	Scotophilus_kuhlii	0	NA	NA	0	1	FILO-L	1	NA	NA	Wacharapluesadee et al. 2015	Used consensus primer (FILO-L) for all filoviruses
universal	Taphozous_longimanus	0	NA	NA	0	27	FILO-L	27	NA	NA	Wacharapluesadee et al. 2015	Used consensus primer (FILO-L) for all filoviruses
universal	Taphozous_melanopogon	0	NA	NA	0	110	FILO-L	110	NA	NA	Wacharapluesadee et al. 2015	Used consensus primer (FILO-L) for all filoviruses
LLOV	Miniopterus_schreibersii	0	NA	NA	20	70	FILO-L	70	NA	NA	Negredo et al. 2011	PCR tested only (specialized filoviral L, and normal FILO-L, and FILO-N). 25 carcasses collected **during** die-offs. 1295 bats later collected negative, not reported by species other than 45 negative schreibersii
LLOV	Myotis_myotis	0	NA	NA	0	9	FILO-L	9	NA	NA	Negredo et al. 2011	PCR tested only (specialized filoviral L, and normal FILO-L, and FILO-N). 9 carcasses collected during die-offs.
	Rousettus_leschenaultii	1	NA	NA	1	29	FILO-L	29	NA	NA	He et al. 2015	Filoviral primer returned virus with previously-unseen sequence; clusters with Ebolaviruses, basal to the EBOV/LLOV clade.
ZEBOV	Acerodon_jubatus	1	1	56	NA	NA	NA	56	NA	NA	Jayme et al. 2015	Screened 1:1, came back more specifically positive.
RESTV	Acerodon_jubatus	1	2	56	0	56	RESTV-NP	56	NA	NA	Jayme et al. 2015	Screened 1:1, came back more specifically positive.
ZEBOV	Pteropus_vampyrus	1	0	5	NA	NA	NA	5	NA	NA	Jayme et al. 2015	Screened 1:1, came back more specifically positive.
RESTV	Pteropus_vampyrus	1	1	5	0	5	RESTV-NP	5	NA	NA	Jayme et al. 2015	Screened 1:1, came back more specifically positive.
RESTV	Miniopterus_schreibersii	0	0	44	3	44	RESTV-NP	44	NA	NA	Jayme et al. 2015	Screened 1:1, came back Ab-. At most 44 indivisduals tested; not all abts had all samples taken and authors did not specify.

Amman, B. R., et al. (2012a), 'Seasonal pulses of Marburg virus circulation in juvenile Rousettus aegyptiacus bats coincide with periods of increased risk of human infection', Plos Pathogens, 8 (10).
Angell, Ruth L., Butlin, Roger K., and Altringham, John D. (2013), 'Sexual Segregation and Flexible Mating Patterns in Temperate Bats', Plos One, 8 (1).
Bergmans, W. (1979), 'Taxonomy and zoogeography of the fruit bats of the Peoples Republic of Congo, with notes on their reproductive biology (Mammalia, Megachiroptera)', Bijdragen Tot De Dierkunde, 48 (2), 161-86.
Bradbury, J. W. (1977), 'Lek mating-behavior in hammer-headed bat', Zeitschrift fur Tierpsychologie-Journal of Comparative Ethology, 45 (3), 225-55.
Breman, J. G., et al. (1999), 'A Search for Ebola Virus in Animals in the Democratic Republic of the Congo and Cameroon: Ecologic, Virologic, and Serologic Surveys, 1979-1980', J Infect Dis, 179 Suppl 1, S137-147.
Brosset, A. and Girons, H. S. (1980), 'Breeding cycles of the microchrioptera living in caves in north eastern Gaboon', Mammalia, 44 (2), 225-32.
Cotterill, F. P. D. and Fergusson, R. A. (1999), 'Reproductive ecology of Commerson's leaf-nosed bats Hipposideros commersoni (Chiroptera : Hipposideridae) in South-Central Africa: interactions between seasonality and large body size; and implications for conservation ', South African Journal of Zoology, 34 (2), 53-63.
Deanesly, R. (1939), 'Observations on pregnancy in the common bat (Pipistrellus pipistrellus)', Proceedings of the Zoological Society of London Series a-General and Experimental, 109, 57-U12.
Francis, C., et al. (2914), 'Pipistrellus javanicus', IUCN Red List of Threatened Species 29 May.
Furey, Neil M., Mackie, Iain J., and Racey, Paul A. (2011), 'Reproductive phenology of bat assemblages in Vietnamese karst and its conservation implications', Acta Chiropterologica, 13 (2), 341-54.
Germain, M. (1978), 'Collection of mammals and arthropods during the epidemic of haemorrhagic fever in Zaire. Ebola virus haemorrhagic fever', in: Pattyn S, editor, Amsterdam, Netherlands: Elsevier/North-Holland Biomedical Press, 185-9.
Gould, E. (1978), 'Rediscovery of Hipposideros ridleyi and seasonal reproduction in Malaysian bats', Biotropica, 10 (1), 30-32.
Hayman, D. T. S., et al. (2012b), 'Demography of straw-colored fruit bats in Ghana', Journal of Mammalogy, 93 (5), 1393_404.
Hayman, D. T., et al. (2010), 'Long-term survival of an urban fruit bat seropositive for Ebola and Lagos bat viruses', PLoS One, 5 (8), e11978.
Hayman, D. T., et al. (2012a), 'Ebola virus antibodies in fruit bats, Ghana, West Africa', Emerg Infect Dis, 18 (7), 1207-9.
He, Biao, Yun Feng, Hailin Zhang, Lin Xu, Weihong Yang, Yuzhen Zhang, Xingyu Li, and Changchun Tu. "Filovirus RNA in Fruit Bats, China." *Emerging Infectious Diseases* 21, no. 9 (September 2015). doi:10.3201/eid2109.150260.

Heideman, P. D. (1989), 'Delayed development in Fischer pygmy fruit bat, Haplonycteris fischeri, in the Philippines', Journal of Reproduction and Fertility, 85 (2), 363-82.
Heideman, P. D. and Heaney, L. R. (1989), 'Population biology and estimates of abundance of fruit bats (Pteropodidae) in Philippine submontane rainforest', Journal of Zoology, 218, 565-86.
Heideman, P. D. and Powell, K. S. (1998), 'Age-specific reproductive strategies and delayed embryonic development in an old world fruit bat, Ptenochirus jagori', Journal of Mammalogy, 79 (1), 295-311.
Heideman, P. D. and Utzurrum, R. C. (2003a), 'Seasonality and synchrony of reproduction in three species of nectarivorous Philippines bats', BMC Ecol, 3, 11.
Heideman, Paul D. and Utzurrum, Ruth C. B. (2003b), 'Seasonality and synchrony of reproduction in three species of nectarivorous Philippines bats', BMC Ecology, 3 (11 Cited January 8, 2004).
Hickey, M. B. C. and Dunlop, J. M. (2000), 'Nycteris grandis', Mammalian Species, (632), 1-4.
Ho, Y. Y. and Lee, L. L. (2003), 'Roost selection by Formosan leaf-nosed bats (Hipposideros armiger terasensis)', Zoolog Sci, 20 (8), 1017-24.
Jayme, Sarah I., Hume E. Field, Carol de Jong, Kevin J. Olival, Glenn Marsh, Anson M. Tagtag, Tom Hughes, et al. “Molecular Evidence of Ebola Reston Virus Infection in Philippine Bats.” *Virology Journal* 12, no. 1 (July 17, 2015): 107. doi:10.1186/s12985-015-0331-3.
Jin, Longru, et al. (2010), 'Postnatal growth and age estimation in the ashy leaf-nosed bat, Hipposideros cineraceus', Acta Chiropterologica, 12 (1), 155-60.
Krishna, A. and Dominic, C. J. (1983), 'Reproduction in the female short-nosed fruit bat Cynopterus sphinx Vahl', Periodicum Biologorum, 85 (1), 23-30.
Langevin, P. and Barclay, R. (1990), 'Hypsignathus monstrosus', Mammalian Species, 357, 1-4.
Leirs, et al. (1999), 'Search for the Ebola virus reservoir in Kikwit, Democratic Republic of the Congo: reflections on a vertebrate collection', J Infect Dis, 179 Suppl 1, S155-S163.
Leroy, E. M., et al. (2005), 'Fruit bats as reservoirs of Ebola virus', Nature, 438 (7068), 575-6.
Lin, A. Q., et al. (2011), 'Postnatal development in Andersen's leaf-nosed bat Hipposideros pomona: flight, wing shape, and wing bone lengths', Zoology (Jena), 114 (2), 69-77.
Lin, Ai-Qing, et al. (2010), 'Postnatal growth and age estimation in Horsfield's leaf-nosed bat Hipposideros larvatus', Zoological Studies, 49 (6), 789-96.
Medway, L. (1971), 'Observations of social and reproductive biology of bent winged bat Miniopterus australis in northern Borneo', Journal of Zoology, 165 (OCT), 261-&.
Negredo, Ana, Gustavo Palacios, Sonia Vázquez-Morón, Félix González, Hernán Dopazo, Francisca Molero, Javier Juste, et al. “Discovery of an Ebolavirus-like Filovirus in Europe.” *PLoS Pathog* 7, no. 10 (2011): e1002304.
Nowak, R. M., Kunz, T. H., and Pierson, E. D. (1994), Walker's Bats of the World (Baltimore: The Johns Hopkins University Press).
Ogawa, Hirohito, Hiroko Miyamoto, Eri Nakayama, Reiko Yoshida, Ichiro Nakamura, Hirofumi Sawa, Akihiro Ishii, et al. “Seroepidemiological Prevalence of Multiple Species of Filoviruses in Fruit Bats (Eidolon Helvum) Migrating in Africa.” *The Journal of Infectious Diseases* 212 Suppl 2 (October 1, 2015): S101-108. doi:10.1093/infdis/jiv063.
Okia, N. O. (1974), 'Breeding in Franquet's bat, Epomops franquetti (Tomes), in Uganda', J Mammal, 55 (2), 462-5.
Olival, K. J., et al. (2013), 'Ebola virus antibodies in fruit bats, bangladesh', Emerg Infect Dis, 19 (2), 270-3.
Pourrut, X., et al. (2007), 'Spatial and temporal patterns of Zaire ebolavirus antibody prevalence in the possible reservoir bat species', J Infect Dis, 196 Suppl 2, S176-83.
Pourrut, X., et al. (2009), 'Large serological survey showing cocirculation of Ebola and Marburg viruses in Gabonese bat populations, and a high seroprevalence of both viruses in Rousettus aegyptiacus', BMC Infect Dis, 9, 159.
Richardson, E. G. (1977), 'Biology and evolution of reproductive cycle of Miniopterus schreibersii and M australis (Chiroptera Vespertilionidae)', Journal of Zoology, 183 (NOV), 353-75.
Rossiter, S. J., et al. (2005), 'Mate fidelity and intra-lineage polygyny in greater horseshoe bats', Nature, 437 (7057), 408-11.
Saéz, A.M., et al. (2015), 'Investigating the zoonotic origin of the West African Ebola epidemic', EMBO molecular medicine, 7 (1), 17-23.
Sandhu, S. and Gopalakrishna, A. (1984), 'Some observations on the breeding biology of the Indian fruit bat, Cynopterus sphinx (Vahl) in central India', Current Science, 53 (22), 1189-92.
Swanepoel, R., et al. (2007), 'Studies of reservoir hosts for Marburg virus', Emerging Infectious Diseases, 13 (12), 1847-51.
Taniguchi, S., et al. (2011), 'Reston ebolavirus antibodies in bats, the Philippines', Emerg Infect Dis, 17 (8), 1559-60.
Thomas, D. W. and Marshall, A. G. (1984), 'Reproduction and growth in 3 species of West African fruit bats', Journal of Zoology, 202 (FEB), 265-81.
Towner, J. S., et al. (2007), 'Marburg virus infection detected in a common African bat', PLoS One, 2 (1), e764.
Towner, J. S., et al. (2009), 'Isolation of genetically diverse Marburg viruses from Egyptian fruit bats', PLoS Pathogens, 5 (7), e1000536.
Wacharapluesadee, Supaporn, Kevin J. Olival, Budsabong Kanchanasaka, Prateep Duengkae, Supakarn Kaewchot, Phimchanok Srongmongkol, Gittiyaporn Ieamsaard, et al. “Surveillance for Ebola Virus in Wildlife, Thailand.” *Emerging Infectious Diseases* 21, no. 12 (December 2015): 2271–73. doi:10.3201/eid2112.150860.
Wright, G. S. (2009), 'Hipposideros caffer (Chiroptera: Hipposideridae)', Mammalian Species, 845, 1-9.
Yuan, J., et al. (2012), 'Serological evidence of ebolavirus infection in bats, China', Virol J, 9, 236.
Zubaid, A. and Davison, G. W. H. (1987), 'A comparative study of the baculum in peninsular Malaysian Hipposiderines', Mammalia, 51 (1), 139-44.
