# Supplementary material for: Undiscovered Bat Hosts of Filoviruses
Source: PLoS Negl Trop Dis. 2016 Jul 14;10(7):e0004815. doi: 10.1371/journal.pntd.0004815 (PMC4945033; doi:10.1371/journal.pntd.0004815)
Supplement: S2 Table — Coverage is calculated as a percentage of the total bat species (out of N = 1116) and a percentage of the total filovirus-positive bat species (out of N = 21) for which there were data available for a given variable. (PDF) [file pntd.0004815.s003.pdf]

	Predictor	% Coverage for all bats	% Coverage for filo+ bats	Definition	Units
2	Activity Cycle	6.63	23.81	Activity cycle of each species measured for non-captive populations; adult or age unspecified individuals, male, female, or sex unspecified individuals; primary, secondary, or extrapolated sources; all measures of central tendency; in all localities. Species were defined as (1) nocturnal only, (2) nocturnal/crepuscular, cathemeral, crepuscular or diurnal/crepuscular and (3) diurnal only.	NA
3	Adult Body Mass (g)	62.28	95.24	Mass of adult (or age unspecified) live or freshly-killed specimens (excluding pregnant females) using captive, wild, provisioned, or unspecified populations; male, female, or sex unspecified individuals; primary, secondary, or extrapolated sources; all measures of central tendency; in all localities.	grams
4	Adult Forearm Length (mm)	80.56	100.00	Total length from elbow to wrist of adult (or age unspecified) live, freshly-killed, or museum specimens using captive, wild, provisioned, or unspecified populations; male, female, or sex unspecified individuals; primary, secondary, or extrapolated sources; all measures of central tendency; in all localities.	mm
5	Adult Head Body Length (mm)	14.16	47.62	Total length from tip of nose to anus or base of tail of adult (or age unspecified) live, freshly-killed, or museum specimens using captive, wild, provisioned, or unspecified populations; male, female, or sex unspecified individuals; primary, secondary, or extrapolated sources; all measures of central tendency; in all localities.	mm
6	Age at Eye Opening (d)	4.03	14.29	Age at which both eyes are fully open after birth using captive, wild, provisioned, or unspecified populations; male, female, or sex unspecified individuals; primary, secondary, or extrapolated sources; all measures of central tendency; in all localities.	days
7	Age at First Birth (d)	4.75	28.57	Age at which females give birth to their first litter (eutherians), or their young attach to teats (metatherians) or hatch out (monotremes), using non-captive, wild, provisioned, or unspecified populations; primary, secondary, or extrapolated sources; all measures of central tendency; in all localities.	days
8	Basal Metabolic Rate (mLO2/hr)	7.17	9.52	Basal metabolic rate of adult (or age unspecified) individual(s) using captive, wild, provisioned, or unspecified populations; male, female, or sex unspecified individuals; primary, secondary, or extrapolated sources; all measures of central tendency; in all localities. Metabolic rate was measured when individual(s) were experiencing neither heat nor cold stress (i.e. are in their thermoneutral zone); are resting and calm; and are post–absorptive (are not digesting or absorbing a meal) and data were only accepted where there was also a measure of body mass for the same individual(s).	mLO2/hr
9	Basal Metabolic Rate Per Gram Mass (g)	7.17	9.52	Mass of individual(s) from which the basal metabolic rate was taken.	grams
10	Diet Breadth	40.50	76.19	Number of dietary categories eaten by each species measured using any qualitative or quantitative dietary measure, over any period of time, using any assessment method, for non-captive or non-provisioned populations; adult or age unspecified individuals, male, female, or sex unspecified individuals; primary, secondary, or extrapolated sources; all measures of central tendency; in all localities. Categories were defined as vertebrate, invertebrate, fruit, flowers/nectar/pollen, leaves/branches/bark, seeds, grass and roots/tubers.	No. of diet categories
11	Gestation Length (d)	13.98	66.67	Length of time of non-inactive fetal growth, using captive, wild, provisioned, or unspecified populations; male, female, or sex unspecified individuals; primary, secondary, or extrapolated sources; all measures of central tendency; in all localities. Gestation was measured between specified start and end points as follows: Start points – conception, fertilization, first observed copulation, fertilization, implantation, laying, palpably pregnant, removal of pouch young, capture (except marsupials) or unspecified. End points – birth, hatching or unspecified.	days
13	Litter Size	41.67	80.95	Number of offspring born per litter per female, either counted before birth, at birth or after birth, using captive, wild, provisioned, or unspecified populations; male, female, or sex unspecified individuals; primary, secondary, or extrapolated sources; all measures of central tendency; in all localities.	no. of offspring
14	Litters Per Year	20.34	71.43	Number of litters per female per year using non-captive, wild, provisioned, or unspecified populations; male, female, or sex unspecified individuals; primary, secondary, or extrapolated sources; all measures of central tendency; in all localities.	no. of litters
15	Max Longevity (m)	6.18	19.05	Maximum adult age measured either through direct observation, capture-recapture estimates, projected from physical wear or unspecified, using captive, wild, provisioned, or unspecified populations; male, female, or sex unspecified individuals; primary, secondary, or extrapolated sources; in all localities.	months

16	Neonate Body Mass (g)	15.14	61.90	Mass of live or freshly-killed specimens of infants at either a near term embryonic stage, birth, immediately after birth or up to an age of seven days after birth, using captive, wild, provisioned, or unspecified populations; male, female, or sex unspecified individuals; primary, secondary, or extrapolated sources; all measures of central tendency; in all localities.	grams
17	Neonate Head Body Length (mm)	7.26	28.57	Total length from tip of nose to anus or base of tail of live, freshly-killed, or museum specimens of infants at either a near term embryonic stage, birth, immediately after birth or up to an age of seven days after birth, using captive, wild, provisioned, or unspecified populations; male, female, or sex unspecified individuals; primary, secondary, or extrapolated sources; all measures of central tendency; in all localities.	mm
18	Population Group Size	9.86	52.38	Number of individuals, adults or definition unspecified in a group (i.e., a roost) that spend the majority of their time in a 24 hour cycle together, measured over any duration of time, using non-captive populations; male, female, or sex unspecified individuals; primary, secondary, or extrapolated sources; all measures of central tendency; in all localities.	no. of individuals, adults or unspecified
19	Sexual Maturity Age (d)	7.89	52.38	Age when individuals are first physically capable of reproducing, defined as either physically sexually mature, age at first mating or unspecified (males and females), age at first estrus or age at first pregnancy (females only), age at spermatogenesis or age at testes descent (males only), using captive, wild, provisioned, or unspecified populations; male, female, or sex unspecified individuals, primary, secondary, or extrapolated sources; all measures of central tendency; in all localities.	days
20	Social Group Size	3.67	19.05	Number of individuals, adults or definition unspecified in a group that spends the majority of their time in a 24 hour cycle together where there is some indication that these individuals form a social cohesive unit, measured over any duration of time, using non-captive populations; male, female, or sex unspecified individuals; primary, secondary, or extrapolated sources; all measures of central tendency; in all localities.	no. of individuals, adults or unspecified
21	Trophic Level	40.50	76.19	Trophic level of each species measured using any qualitative or quantitative dietary measure, over any period of time, using any assessment method, for non-captive or non-provisioned populations; adult or age unspecified individuals, male, female, or sex unspecified individuals; primary, secondary, or extrapolated sources; all measures of central tendency; in all localities. Species were defined as (1) herbivore (not vertebrate and/or invertebrate), (2) omnivore (vertebrate and/or invertebrate plus any of the other categories) and (3) carnivore (vertebrate and/or invertebrate only)	NA
22	Weaning Age (d)	12.54	52.38	Age when primary nutritional dependency on the mother ends and independent foraging begins to make a major contribution to the offspring’s energy requirements, measured as either weaning/lactation length, nutritionally independent, first solid food, last observed nursing, age at first flight (bats only), age at pouch exit or length of teat attachment (marsupials only) or unspecified definition, using captive, wild, provisioned, or unspecified populations; male, female, or sex unspecified individuals; primary, secondary, or extrapolated sources; all measures of central tendency; in all localities.	days
23	Weaning Body Mass (g)	5.20	42.86	Mass of live or freshly-killed specimens of weanlings, using captive, wild, provisioned, or unspecified populations; male, female, or sex unspecified individuals; primary, secondary, or extrapolated sources; all measures of central tendency; in all localities.	grams
25	Adult Body Mass (g) EXT	21.59	4.76	A fitted value for Adult Body Mass calculated by fitting across-species generalized linear models using the species medians of the consolidated values; see Class V.B., Table 3 in PanTHERIA for GLM model details	
27	Geographic Range Area (km2)	95.70	95.24	Calculated using the total extent of a species range with a global equal-area projection (Mollweide)	km2
28	Max Lat of Geographic Range (dd)	95.70	95.24	Maximum latitudinal extent of each species range calculated using a global geographic projection (decimal degrees)	dd
29	Min Latitude of Geographic Range (dd)	95.70	95.24	Minimum latitudinal extent of each species range calculated using a global geographic projection (decimal degrees)	dd
30	Midrange Latitude of Geographic Range (dd)	95.70	95.24	Median latitudinal extent of each species range calculated using a global geographic projection (decimal degrees)	dd
31	Max Longitude of Geographic Range (dd)	95.70	95.24	Maximum longitudinal extent of each species range calculated using a global geographic projection (decimal degrees)	dd
32	Min Longitude of Geographic Range (dd)	95.70	95.24	Minimum longitudinal extent of each species range calculated using a global geographic projection (decimal degrees)	dd

33	Midrange Longitude of Geographic Range (dd)	95.70	95.24	Median longitudinal extent of each species range calculated using a global geographic projection (decimal degrees)	dd
34	Min Human Population Density (n.km2)	95.70	95.24	Minimum human population density (persons per km2) using the Gridded Population of the World (GPW) (CIESIN and CIAT 2005) for 1995	persons per km2
35	Mean Hu Pop Den (n.km2)	95.70	95.24	Mean human population density (persons per km2) using the Gridded Population of the World (GPW) (CIESIN and CIAT 2005) for 1995	persons per km2
36	X27.3_HuPopDen_5p_n.km2 (n.km2)	95.70	95.24	Mean human population density (persons per km2) using the Gridded Population of the World (GPW) (CIESIN and CIAT 2005) for 1995	persons per km2
37	Change in Human Population Density	95.34	95.24	Mean rate of increase in human population density using the Gridded Population of the World (GPW) (CIESIN and CIAT 2005) for 1990 and 1995 as: (1995–1990)/1990	
38	Mean Precipitation (mm)	91.13	95.24	Mean monthly precipitation (mm) calculated using data from ftp://ftp.ngdc.noaa.gov/Solid_Earth/Ecosystems/GEDII_a/datasets/a03/lc.htm	mm
39	Mean Temp (01degC)	91.13	95.24	Mean monthly temperature (0.1°C) calculated using data from ftp://ftp.ngdc.noaa.gov/Solid_Earth/Ecosystems/GEDII_a/datasets/a03/lc.htm	degrees C
40	Mean AET (mm)	82.97	95.24	Mean monthly AET (Actual Evapotranspiration Rate) from 1920 to 1980 (mm) calculated using the Global Resource Information Database of UNEP and is available from http://www.grid.unep.ch/data/grid/gnv183.php	mm
41	Mean PET (mm)	82.97	95.24	Mean monthly PET (Potential Evapotranspiration Rate) from 1920 to 1980 (mm) calculated using the Global Resource Information Database of UNEP and is available from http://www.grid.unep.ch/data/grid/gnv183.php	mm
42	Log10 Production	4.48	42.86	The log-base 10 of production, where production is the mean mass of offspring produced per year, normalized by adult body size	
43	WOS Citation Count	100.00	100.00	Number of citations returned by a Web of Science search on bat species binomials	
44	Diet Invertebrates	99.91	100.00	Percent of diet comprised of invertebrates	
45	Diet Fruit	99.91	100.00	Percent of diet comprised of fruit	
46	ForStrat.Value	99.91	100.00	Assignment to one of five foraging stratum categories	
47	BodyMass.Value (g)	99.91	100.00	Mean of body mass for both sexes (in g)	grams
48	Torpor	5.73	38.10	Categorization of torpor use, where 1 = no evidence of torpor use; 2 = some torpor use with minimum body temperature> 11◦C; 3 = true hibernation with min Tb< 11◦C	
49	Migratory	5.73	38.10	Categorization of migration level, where 1 = only local movement; 2 = regional migration, 3 = long distance migration	
50	IUCN	5.73	38.10	IUCN conservation status	
51	Aridity	82.97	95.24	Mean Precipitation (mm)/Mean PET (mm)	
52	BodySizeRatio	15.14	61.90	Adult Body Mass (g)/Neonate Body Mass (g)	
53	Postnatal Growth Rate	4.39	28.57	Weaning body mass/neonatal body mass	
54	Relative Age at Sexual Maturity	3.41	14.29	Age at sexual maturity/maximum longevity	
55	Mammalian Species Density (n/km2)	88.26	85.71	The richness of mammal species found within a species' geographic range divided by the geographic range area in units of n per kilometer2	species per km2
