# Supplementary material for: Undiscovered Bat Hosts of Filoviruses
Source: PLoS Negl Trop Dis. 2016 Jul 14;10(7):e0004815. doi: 10.1371/journal.pntd.0004815 (PMC4945033; doi:10.1371/journal.pntd.0004815)
Supplement: S3 Table — The first 112 species comprise the 90th percentile probability of novel filovirus-positive bat species. Label is a binomial variable denoting filovirus-positivity. Probability is a transformation of model outputs. (PDF) [file pntd.0004815.s004.pdf]

Species	output	label	probability
Rousettus_aegyptiacus	0.90642502	1	0.71226806
Cynopterus_sphinx	0.83001727	1	0.69635858
Rousettus_leschenaultii	0.78840058	1	0.6874878
Rousettus_amplexicaudatus	0.78618184	1	0.68701091
Epomops_franqueti	0.73951351	1	0.67688947
Miniopterus_schreibersii	0.58358838	1	0.64189267
Hypsignathus_monstrosus	0.5633075	1	0.63721749
Pipistrellus_pipistrellus	0.51764116	1	0.62659602
Mops_condylurus	0.43884416	1	0.60798358
Miniopterus_inflatus	0.30201942	1	0.57493611
Myonycteris_torquata	0.28886619	1	0.57171854
Artibeus_lituratus	0.28006534	0	0.56956224
Eidolon_helvum	0.26493532	1	0.56584911
Rhinolophus_affinis	0.25408387	1	0.56318143
Scotophilus_kuhlii	0.19338504	1	0.54819615
Miniopterus_pusillus	0.1176852	1	0.52938739
Rhinolophus_pusillus	0.11315528	0	0.52825867
Pipistrellus_tenuis	0.0976695	0	0.52439798
Tadarida_brasiliensis	0.09272254	0	0.52316404
Eonycteris_spelaea	0.08844296	0	0.52209634
Myotis_ricketti	0.08596453	1	0.52147791
Megaderma_lyra	0.08139516	0	0.52033756
Myotis_lucifugus	0.08097215	0	0.52023199
Hipposideros_pomona	0.07853223	1	0.51962297
Rhinolophus_ferrumequinum	0.0783308	0	0.51957269
Phyllostomus_hastatus	0.07603956	0	0.51900074
Hipposideros_commersoni	0.07337517	0	0.51833557
Epomophorus_labiatus	0.07002816	0	0.51749989
Pteropus_livingstonii	0.06337008	0	0.51583722
Rhinolophus_monoceros	0.06226401	0	0.51556097
Carollia_perspicillata	0.06115001	0	0.51528274
Hipposideros_armiger	0.05969518	0	0.51491937
Myotis_adversus	0.05799721	0	0.51449524
Chaerephon_pumilus	0.05206168	0	0.51301248
Rhinolophus_lepidus	0.05164175	0	0.51290757
Artibeus_jamaicensis	0.04959917	0	0.51239725
Dobsonia_magna	0.04758663	0	0.51189441
Micropteropus_pusillus	0.04738362	1	0.51184369
Otomops_martiensseni	0.04565115	0	0.51141081
Tylonycteris_robustula	0.04557026	0	0.5113906
Hypsugo_savii	0.04476219	0	0.51118868

Saccolaimus_saccolaimus	0.04249051	0	0.51062103
Rhinolophus_euryale	0.04095819	0	0.51023812
Pteropus_rodricensis	0.0394427	0	0.5098594
Miniopterus_australis	0.03919412	0	0.50979728
Ptenochirus_jagori	0.03905807	0	0.50976328
Dobsonia_moluccensis	0.03875868	0	0.50968846
Pteropus_conspicillatus	0.03816896	0	0.50954108
Epomops_buettikoferi	0.03743002	0	0.50935641
Pipistrellus_abramus	0.03621884	0	0.50905372
Pteropus_ornatus	0.03590386	0	0.508975
Platyrrhinus_lineatus	0.03569601	0	0.50892306
Rhinolophus_sinicus	0.03494929	0	0.50873643
Scotophilus_heathii	0.03361392	0	0.50840269
Myotis_albescens	0.0332037	0	0.50830016
Hipposideros_gigas	0.03298889	0	0.50824648
Pteropus_giganteus	0.03040236	0	0.50760001
Murina_leucogaster	0.03010533	0	0.50752577
Epomophorus_gambianus	0.02921073	1	0.50730216
Pteropus_pumilus	0.02811183	0	0.50702749
Rhinolophus_eloquens	0.02806231	1	0.50701512
Molossus_molossus	0.02798127	0	0.50699486
Nanonycteris_veldkampii	0.02796134	1	0.50698988
Brachyphylla_cavernarum	0.02695011	0	0.50673712
Hipposideros_bicolor	0.02599054	0	0.50649727
Myotis_fimbriatus	0.025055	0	0.50626342
Myotis_myotis	0.02458106	0	0.50614496
Rhinolophus_luctus	0.02404223	0	0.50601027
Rhinolophus_cornutus	0.02359982	0	0.50589968
Myotis_velifer	0.02295538	0	0.50573859
Pteropus_melanotus	0.02267315	0	0.50566805
Pteropus_scapulatus	0.02261674	0	0.50565394
Cynopterus_brachyotis	0.02243074	0	0.50560745
Dobsonia_praedatrix	0.02236608	0	0.50559129
Peropteryx_kappleri	0.02185091	0	0.50546251
Coleura_afra	0.02182309	0	0.50545556
Eonycteris_major	0.02165709	0	0.50541406
Taphozous_nudiventris	0.02155931	0	0.50538962
Pteropus_tonganus	0.02136416	0	0.50534084
Rhinolophus_acuminatus	0.02080617	0	0.50520136
Rhinolophus_macrotis	0.02049502	0	0.50512358
Mops_midas	0.02039519	0	0.50509862
Nyctalus_lasiopterus	0.02033287	0	0.50508304

Rhinolophus_shortridgei	0.02024302	0	0.50506058
Pteropus_dasymallus	0.02024058	0	0.50505997
Tylonycteris_pachypus	0.02012274	0	0.50503051
Phyllostomus_discolor	0.01982018	0	0.50495488
Pteropus_poliocephalus	0.01966413	0	0.50491587
Hipposideros_speoris	0.01966386	0	0.50491581
Artibeus_phaeotis	0.01943315	0	0.50485813
Myotis_formosus	0.01925009	0	0.50481237
Myotis_tricolor	0.01858738	0	0.50464671
Macroglossus_minimus	0.01815186	0	0.50453784
Megaderma_spasma	0.01806531	0	0.5045162
Sturnira_aratathomasi	0.01793359	0	0.50448328
Macroderma_gigas	0.01761412	0	0.50440342
Artibeus_aztecus	0.01761046	0	0.5044025
Corynorhinus_rafinesquii	0.01759461	0	0.50439854
Desmodus_rotundus	0.01758948	0	0.50439726
Rhinopoma_microphyllum	0.01726881	0	0.5043171
Coelops_frithii	0.01720833	0	0.50430198
Pteropus_rayneri	0.01709237	0	0.50427299
Eptesicus_fuscus	0.01707912	0	0.50426968
Taphozous_melanopogon	0.01699819	0	0.50424944
Brachyphylla_nana	0.01695387	0	0.50423837
Chaerephon_plicatus	0.01689168	0	0.50422282
Taphozous_perforatus	0.01683223	0	0.50420796
Eumops_perotis	0.01672363	0	0.50418081
Rhinolophus_maendeleo	0.0166908	0	0.5041726
Rhinolophus_pearsonii	0.01667156	0	0.50416779
Harpiocephalus_harpia	0.01665704	0	0.50416416
Pipistrellus_ceylonicus	0.01662606	0	0.50415642
Glossophaga_soricina	0.01656478	0	0.5041411
Epomophorus_wahlbergi	0.0163717	0	0.50409283
Pipistrellus_coromandra	0.01628393	0	0.50407089
Pteropus_vampyrus	0.01617462	0	0.50404357
Phoniscus_jagorii	0.01561271	0	0.5039031
Taphozous_longimanus	0.01553617	0	0.50388396
Rhinolophus_clivosus	0.01540937	0	0.50385227
Murina_cyclotis	0.01530987	0	0.50382739
Glossophaga_commissarisi	0.01528603	0	0.50382143
Platyrrhinus_helleri	0.01525165	0	0.50381284
Scotophilus_leucogaster	0.01490418	0	0.50372598
Hipposideros_ater	0.01454802	0	0.50363694
Myotis_macrodactylus	0.01452717	0	0.50363173

Rhinolophus_blasii	0.01435204	0	0.50358795
Lasiurus_ega	0.01427851	0	0.50356957
Tadarida_teniotis	0.01415396	0	0.50353843
Pteropus_howensis	0.0141146	0	0.50352859
Hipposideros_caffer	0.01408971	0	0.50352237
Myotis_blythii	0.01408741	0	0.5035218
Cardioderma_cor	0.01406157	0	0.50351533
Philetor_brachypterus	0.01378766	0	0.50344686
Pipistrellus_kuhlii	0.01374582	0	0.5034364
Lasiurus_blossevillii	0.01372205	0	0.50343046
Lophostoma_silvicolum	0.01354938	0	0.50338729
Myotis_siligorensis	0.01349915	0	0.50337474
Kerivoula_picta	0.01343874	0	0.50335963
Uroderma_bilobatum	0.01340375	0	0.50335089
Rhinolophus_madurensis	0.01339126	0	0.50334776
Rhinolophus_trifoliatus	0.01338065	0	0.50334511
Tadarida_fulminans	0.01335803	0	0.50333946
Rhinolophus_beddomei	0.01333168	0	0.50333287
Rhinolophus_mehelyi	0.01331189	0	0.50332792
Eidolon_dupreanum	0.01331023	0	0.50332751
Carollia_brevicauda	0.01309859	0	0.5032746
Rhinolophus_imaizumii	0.01305115	0	0.50326274
Pipistrellus_subflavus	0.01303183	0	0.50325791
Myotis_horsfieldii	0.01292253	0	0.50323059
Hipposideros_diadema	0.01289403	0	0.50322346
Nycteris_macrotis	0.01288477	0	0.50322115
Nycteris_thebaica	0.01278111	0	0.50319523
Eptesicus_furinalis	0.01274565	0	0.50318637
Hypsugo_alaschanicus	0.01272799	0	0.50318196
Myotis_hosonoi	0.01272097	0	0.5031802
Rhinolophus_canuti	0.01267647	0	0.50316908
Rhinolophus_philippinensis	0.0126573	0	0.50316428
Murina_grisea	0.01252779	0	0.50313191
Ia_io	0.01244721	0	0.50311176
Myotis_leibii	0.01237505	0	0.50309372
Lasionycteris_noctivagans	0.01226959	0	0.50306736
Thyroptera_tricolor	0.01226025	0	0.50306503
Rhinolophus_capensis	0.01220506	0	0.50305123
Hypsugo_imbricatus	0.01209846	0	0.50302458
Hipposideros_halophyllus	0.01206591	0	0.50301644
Glossophaga_longirostris	0.01200603	0	0.50300147
Rhinolophus_formosae	0.01197421	0	0.50299352

Lasiurus_borealis	0.01187645	0	0.50296908
Nyctalus_noctula	0.0118426	0	0.50296062
Hipposideros_larvatus	0.01181797	0	0.50295446
Rhinolophus_simulator	0.01179917	0	0.50294976
Eptesicus_dimissus	0.01177863	0	0.50294462
Pipistrellus_nathusii	0.01173536	0	0.50293381
Rhinolophus_stheno	0.01171921	0	0.50292977
Rhinolophus_arcuatus	0.01168223	0	0.50292052
Rhinopoma_hardwickii	0.01156454	0	0.5028911
Myotis_oxygnathus	0.01149333	0	0.5028733
Miniopterus_fraterculus	0.01143407	0	0.50285849
Falsistrellus_affinis	0.01136546	0	0.50284133
Myotis_ozensis	0.01132656	0	0.50283161
Rhinolophus_celebensis	0.01132205	0	0.50283048
Hipposideros_ruber	0.0113203	0	0.50283005
Nycteris_grandis	0.01129106	0	0.50282274
Trachops_cirrhosus	0.01127662	0	0.50281913
Myotis_pequinius	0.01124258	0	0.50281062
Sturnira_bidens	0.01122435	0	0.50280606
Diaemus_youngi	0.01120203	0	0.50280048
Murina_silvatica	0.01116655	0	0.50279161
Otomops_formosus	0.01108016	0	0.50277001
Harpyionycteris_whiteheadi	0.01105381	0	0.50276342
Molossus_rufus	0.01104633	0	0.50276155
Rhinolophus_rex	0.01104457	0	0.50276111
Rhinolophus_alcyone	0.01103469	0	0.50275865
Latidens_salimalii	0.01101691	0	0.5027542
Scotophilus_dinganii	0.01099632	0	0.50274905
Macrophyllum_macrophyllum	0.01098955	0	0.50274736
Myotis_oreias	0.01095712	0	0.50273925
Hipposideros_cineraceus	0.01091533	0	0.50272881
Eptesicus_brasiliensis	0.01090908	0	0.50272724
Pteronotus_parnellii	0.0109058	0	0.50272642
Histiotus_montanus	0.01088504	0	0.50272123
Micronycteris_microtis	0.01086963	0	0.50271738
Rhinolophus_hilli	0.01083804	0	0.50270948
Pteropus_pselaphon	0.01083194	0	0.50270796
Carollia_castanea	0.01078056	0	0.50269511
Pteronotus_personatus	0.01076159	0	0.50269037
Artibeus_obscurus	0.01075713	0	0.50268926
Myotis_montivagus	0.01073113	0	0.50268276
Eptesicus_platyops	0.01071016	0	0.50267751

Artibeus_toltecus	0.01070905	0	0.50267724
Rhinophylla_pumilio	0.01069055	0	0.50267261
Cynopterus_titthaecheilus	0.01068989	0	0.50267245
Myotis_nigricans	0.01068254	0	0.50267061
Micronycteris_schmidtorum	0.01067725	0	0.50266929
Leptonycteris_yerbabuenae	0.01067187	0	0.50266794
Pteropus_lylei	0.01065083	0	0.50266268
Hipposideros_galeritus	0.01061516	0	0.50265376
Stenoderma_rufum	0.01060073	0	0.50265016
Taphozous_theobaldi	0.01058577	0	0.50264642
Rhinolophus_keyensis	0.01058458	0	0.50264612
Myotis_pruinosus	0.01055622	0	0.50263903
Pipistrellus_endoi	0.01055622	0	0.50263903
Nyctalus_plancyi	0.01055567	0	0.50263889
Dobsonia_peronii	0.01051748	0	0.50262935
Miniopterus_magnater	0.01051298	0	0.50262822
Scotoecus_pallidus	0.01050005	0	0.50262499
Pipistrellus_javanicus	0.01047876	0	0.50261967
Eptesicus_japonensis	0.01047648	0	0.5026191
Asellia_tridens	0.01045785	0	0.50261444
Coleura_seychellensis	0.01045476	0	0.50261367
Lissonycteris_angolensis	0.01043108	0	0.50260775
Sturnira_ludovici	0.01043032	0	0.50260756
Tadarida_aegyptiaca	0.01041934	0	0.50260481
Sturnira_lilium	0.01041146	0	0.50260284
Barbastella_leucomelas	0.01039402	0	0.50259848
Chrotopterus_auritus	0.0103753	0	0.5025938
Molossops_neglectus	0.0103749	0	0.5025937
Peropteryx_macrotis	0.01035586	0	0.50258894
Noctilio_albiventris	0.01035133	0	0.50258781
Nyctalus_aviator	0.0103076	0	0.50257688
Rhinolophus_mitratus	0.01028947	0	0.50257235
Myotis_muricola	0.01023425	0	0.50255854
Kerivoula_hardwickii	0.01020295	0	0.50255072
Paracoelops_megalotis	0.01019933	0	0.50254981
Hipposideros_nequam	0.01019508	0	0.50254875
Pteropus_alecto	0.01015762	0	0.50253938
Megaerops_kusnotoi	0.01013422	0	0.50253353
Rhinolophus_euryotis	0.01008492	0	0.50252121
Rhinolophus_malayanus	0.01008488	0	0.5025212
Myotis_californicus	0.0100612	0	0.50251528
Noctilio_leporinus	0.01000133	0	0.50250031

Myotis_ciliolabrum	0.01000128	0	0.5025003
Centurio_senex	0.00999907	0	0.50249975
Syconycteris_australis	0.00997902	0	0.50249473
Nycteris_javanica	0.00994607	0	0.5024865
Rhinolophus_subrufus	0.00994423	0	0.50248604
Arielulus_circumdatus	0.00993778	0	0.50248442
Hipposideros_sorenseni	0.0099293	0	0.5024823
Hipposideros_madurae	0.00990564	0	0.50247639
Mimon_crenulatum	0.00988382	0	0.50247093
Myotis_alcathoe	0.00985678	0	0.50246417
Scotonycteris_zenkeri	0.00984827	0	0.50246205
Pipistrellus_permixtus	0.00984281	0	0.50246068
Lasiurus_egregius	0.00983506	0	0.50245875
Arielulus_torquatus	0.00982325	0	0.50245579
Plecotus_taivanus	0.00982325	0	0.50245579
Murina_puta	0.00979324	0	0.50244829
Myotis_mystacinus	0.00978835	0	0.50244707
Macrotus_californicus	0.00977943	0	0.50244484
Nyctinomops_macrotis	0.00974189	0	0.50243545
Acerodon_jubatus	0.00973159	0	0.50243288
Macroglossus_sobrinus	0.00972865	0	0.50243214
Glischropus_javanus	0.00971114	0	0.50242777
Falsistrellus_mordax	0.00971107	0	0.50242775
Vampyrum_spectrum	0.0097031	0	0.50242576
Mops_mops	0.00967439	0	0.50241858
Eptesicus_serotinus	0.00966228	0	0.50241555
Molossops_temminckii	0.00966109	0	0.50241525
Rhinolophus_osgoodi	0.00965761	0	0.50241438
Carollia_sowelli	0.00964262	0	0.50241064
Tadarida_insignis	0.00963952	0	0.50240986
Myotis_martiniquensis	0.00962918	0	0.50240728
Myotis_riparius	0.00956734	0	0.50239182
Myotis_occultus	0.00955507	0	0.50238875
Myotis_anjouanensis	0.00955403	0	0.50238849
Rhinolophus_rufus	0.00951435	0	0.50237857
Myotis_capaccinii	0.00948697	0	0.50237172
Scotophilus_nigrita	0.00948266	0	0.50237065
Hypsugo_pulveratus	0.00948031	0	0.50237006
Rhinolophus_virgo	0.00947308	0	0.50236825
Thyroptera_discifera	0.0094548	0	0.50236368
Plecotus_austriacus	0.009451	0	0.50236273
Pygoderma_bilabiatum	0.00944259	0	0.50236063

Rhinolophus_fumigatus	0.00938236	0	0.50234557
Miniopterus_medius	0.00932942	0	0.50233234
Kerivoula_smithii	0.00926937	0	0.50231733
Hipposideros_pygmaeus	0.00926769	0	0.50231691
Hypsugo_cadornae	0.0092631	0	0.50231576
Megaerops_niphanae	0.00924435	0	0.50231107
Murina_tenebrosa	0.00921938	0	0.50230483
Rhinolophus_bocharicus	0.00920268	0	0.50230065
Pteropus_niger	0.00917638	0	0.50229408
Pteropus_leucopterus	0.00917406	0	0.5022935
Hipposideros_hypophyllus	0.0091658	0	0.50229143
Lasiurus_cinereus	0.00914147	0	0.50228535
Hipposideros_pratti	0.00913621	0	0.50228404
Vampyrodes_caraccioli	0.00911796	0	0.50227947
Ectophylla_alba	0.00906695	0	0.50226672
Kerivoula_papillosa	0.00906314	0	0.50226577
Dobsonia_inermis	0.00906231	0	0.50226556
Balionycteris_maculata	0.00902621	0	0.50225654
Harpiocephalus_mordax	0.00901665	0	0.50225415
Miniopterus_fuscus	0.00901412	0	0.50225352
Pipistrellus_pygmaeus	0.00901095	0	0.50225272
Chiroderma_villosum	0.00899895	0	0.50224972
Glossophaga_leachii	0.00899512	0	0.50224876
Hesperoptenus_tickelli	0.0089832	0	0.50224578
Micronycteris_megalotis	0.00896624	0	0.50224154
Myotis_ater	0.00895005	0	0.5022375
Hipposideros_orbiculus	0.00894499	0	0.50223623
Rhinolophus_landeri	0.00892593	0	0.50223147
Pipistrellus_hesperus	0.00891684	0	0.50222919
Rousettus_obliviosus	0.0088905	0	0.50222261
Eptesicus_tatei	0.00888682	0	0.50222169
Saccolaimus_flaviventris	0.0088223	0	0.50220556
Ariteus_flavescens	0.0088223	0	0.50220556
Miniopterus_tristis	0.00880416	0	0.50220103
Murina_ryukyuana	0.00873852	0	0.50218462
Myotis_yanbarensis	0.00873852	0	0.50218462
Otonycteris_hemprichii	0.00872289	0	0.50218071
Megaerops_ecaudatus	0.00871158	0	0.50217788
Rhinolophus_inops	0.00869579	0	0.50217393
Rhinolophus_yunanensis	0.00865406	0	0.5021635
Chaerephon_major	0.00863046	0	0.5021576
Hipposideros_cyclops	0.00859675	0	0.50214917

Tadarida_ventralis	0.00857168	0	0.50214291
Hipposideros_fulvus	0.00856547	0	0.50214135
Barbastella_barbastellus	0.00854802	0	0.50213699
Emballonura_alecto	0.00854443	0	0.50213609
Emballonura_monticola	0.00854375	0	0.50213593
Myotis_bechsteinii	0.00853286	0	0.5021332
Antrozous_pallidus	0.00852297	0	0.50213073
Triaenops_persicus	0.00849794	0	0.50212447
Myotis_nattereri	0.00847987	0	0.50211996
Cynopterus_minutus	0.00846364	0	0.5021159
Nyctalus_leisleri	0.00844755	0	0.50211188
Myotis_hasseltii	0.00839813	0	0.50209952
Lophostoma_carrikeri	0.00839548	0	0.50209886
Monophyllus_redmani	0.00835629	0	0.50208906
Pipistrellus_paterculus	0.00830966	0	0.5020774
Myotis_sicarius	0.00830343	0	0.50207585
Chiroderma_doriae	0.00825781	0	0.50206444
Sphaerias_blanfordi	0.00822311	0	0.50205577
Rhinolophus_megaphyllus	0.00821849	0	0.50205461
Megaloglossus_woermanni	0.00817356	0	0.50204338
Scotomanes_ornatus	0.00817063	0	0.50204265
Leptonycteris_nivalis	0.00814699	0	0.50203674
Myotis_chinensis	0.00812643	0	0.5020316
Nyctalus_montanus	0.0081158	0	0.50202894
Eumops_glaucinus	0.00811028	0	0.50202756
Hipposideros_lekaguli	0.00810513	0	0.50202627
Lonchorhina_aurita	0.00809454	0	0.50202362
Tadarida_latouchei	0.00808937	0	0.50202233
Hipposideros_jonesi	0.00806633	0	0.50201657
Diphylla_ecaudata	0.00806174	0	0.50201543
Myotis_longipes	0.0080536	0	0.50201339
Myotis_volans	0.00801064	0	0.50200265
Nyctalus_azoreum	0.00800469	0	0.50200116
Myotis_bombinus	0.00800307	0	0.50200076
Myotis_altarium	0.00799835	0	0.50199958
Pteropus_samoensis	0.00799363	0	0.5019984
Myotis_dasycneme	0.00799293	0	0.50199822
Mimon_cozumelae	0.0079738	0	0.50199344
Anoura_latidens	0.0079689	0	0.50199221
Natalus_tumidirostris	0.00796246	0	0.5019906
Cynopterus_horsfieldii	0.00794275	0	0.50198568
Myotis_macrotarsus	0.00794069	0	0.50198516

Nyctinomops_aurispinosus	0.00791986	0	0.50197995
Myotis_brandtii	0.00790904	0	0.50197725
Pipistrellus_rueppellii	0.00790868	0	0.50197716
Myotis_welwitschii	0.00790603	0	0.5019765
Plecotus_teneriffae	0.00789681	0	0.50197419
Hipposideros_obscurus	0.00788041	0	0.50197009
Pipistrellus_hesperidus	0.00786456	0	0.50196613
Pipistrellus_maderensis	0.00786189	0	0.50196546
Kerivoula_lanosa	0.00784567	0	0.50196141
Pteronotus_gymnonotus	0.00782635	0	0.50195658
Mormoops_megalophylla	0.00781674	0	0.50195418
Myotis_septentrionalis	0.0078161	0	0.50195401
Balantiopteryx_plicata	0.00780846	0	0.50195211
Artibeus_watsoni	0.0078062	0	0.50195154
Nycticeinops_schlieffeni	0.00780194	0	0.50195048
Pteropus_rufus	0.00777721	0	0.50194429
Rhinolophus_paradoxolophus	0.0077755	0	0.50194387
Vespertilio_sinensis	0.00774955	0	0.50193738
Mimon_bennettii	0.00771938	0	0.50192984
Murina_huttoni	0.00771577	0	0.50192893
Mops_thersites	0.00770663	0	0.50192665
Phylloderma_stenops	0.00769736	0	0.50192433
Emballonura_semicaudata	0.00768922	0	0.50192229
Leptonycteris_curasoae	0.00768572	0	0.50192142
Chiroderma_improvisum	0.00767918	0	0.50191978
Murina_aurata	0.00767692	0	0.50191922
Dobsonia_minor	0.00767507	0	0.50191876
Eumops_auripendulus	0.0076649	0	0.50191622
Rhinolophus_thomasi	0.00764228	0	0.50191056
Scotophilus_borbonicus	0.00763779	0	0.50190944
Eonycteris_robusta	0.00763574	0	0.50190893
Eptesicus_guadeloupensis	0.00763135	0	0.50190783
Erophylla_bombifrons	0.00763047	0	0.50190761
Corynorhinus_mexicanus	0.00762575	0	0.50190643
Phyllostomus_elongatus	0.00762091	0	0.50190522
Sturnira_erythromos	0.00761989	0	0.50190496
Glischropus_tylopus	0.00761868	0	0.50190466
Myotis_keenii	0.00761856	0	0.50190463
Hipposideros_lylei	0.00761651	0	0.50190412
Hesperoptenus_blanfordi	0.00761393	0	0.50190347
Myotis_ikonnikovi	0.00761333	0	0.50190332
Lasiurus_minor	0.00760941	0	0.50190234

Natalus_major	0.00760941	0	0.50190234
Myotis_laniger	0.00760225	0	0.50190055
Phyllonycteris_aphylla	0.00760154	0	0.50190038
Taphozous_hamiltoni	0.00759096	0	0.50189773
Mystacina_robusta	0.00758186	0	0.50189546
Glauconycteris_variegata	0.00757494	0	0.50189373
Myotis_bocagii	0.00757494	0	0.50189373
Aethalops_alecto	0.00757468	0	0.50189366
Glauconycteris_argentata	0.00756424	0	0.50189105
Lasiurus_degelidus	0.00756087	0	0.50189021
Natalus_jamaicensis	0.00756069	0	0.50189016
Rhinolophus_ruwenzorii	0.00755959	0	0.50188989
Furipterus_horrens	0.00755722	0	0.5018893
Tonatia_bidens	0.00754592	0	0.50188647
Cormura_brevirostris	0.0075385	0	0.50188462
Rhinolophus_coelophyllus	0.00753748	0	0.50188436
Sturnira_magna	0.00752578	0	0.50188144
Cynomops_abrasus	0.00752455	0	0.50188113
Cynomops_planirostris	0.00752054	0	0.50188013
Platyrrhinus_infuscus	0.00750152	0	0.50187537
Eumops_dabbenei	0.00749721	0	0.50187429
Hipposideros_ridleyi	0.00749599	0	0.50187399
Mimetillus_moloneyi	0.00749362	0	0.5018734
Sturnira_luisi	0.00748628	0	0.50187156
Choeroniscus_minor	0.00746712	0	0.50186677
Molossus_currentium	0.00746672	0	0.50186667
Rhinolophus_subbadius	0.00746434	0	0.50186608
Miniopterus_manavi	0.00745642	0	0.5018641
Myotis_keaysi	0.00745031	0	0.50186257
Myotis_oxyotus	0.00745031	0	0.50186257
Centronycteris_centralis	0.00744763	0	0.5018619
Chiroderma_salvini	0.00744566	0	0.50186141
Sturnira_thomasi	0.00744293	0	0.50186072
Eumops_hansae	0.00744062	0	0.50186015
Nycteris_gambiensis	0.0074361	0	0.50185902
Cyttarops_alecto	0.0074343	0	0.50185857
Glyphonycteris_sylvestris	0.00743164	0	0.5018579
Lichonycteris_obscura	0.00743164	0	0.5018579
Lionycteris_spurrelli	0.00743164	0	0.5018579
Trinycteris_nicefori	0.00743164	0	0.5018579
Lophostoma_brasiliense	0.00743164	0	0.5018579
Micronycteris_hirsuta	0.00743164	0	0.5018579

Rhinolophus_hildebrandtii	0.00742545	0	0.50185635
Eptesicus_diminutus	0.00742315	0	0.50185578
Chiroderma_trinitatum	0.00742268	0	0.50185566
Enchisthenes_hartii	0.00742268	0	0.50185566
Uroderma_magnirostrum	0.00742268	0	0.50185566
Nycteris_hispida	0.00741888	0	0.50185471
Lonchophylla_thomasi	0.00741398	0	0.50185349
Laephotis_wintoni	0.00741111	0	0.50185277
Artibeus_gnomus	0.00740592	0	0.50185147
Cynomops_paranus	0.00740592	0	0.50185147
Rhinolophus_creaghi	0.0073973	0	0.50184932
Tonatia_saurophila	0.00739129	0	0.50184781
Myotis_simus	0.00738937	0	0.50184733
Saccopteryx_leptura	0.00737372	0	0.50184342
Rhogeessa_io	0.00737372	0	0.50184342
Scotophilus_viridis	0.00737002	0	0.5018425
Pteronotus_davyi	0.00736937	0	0.50184233
Lonchophylla_robusta	0.00734735	0	0.50183683
Glyphonycteris_daviesi	0.0073357	0	0.50183392
Cynomops_greenhalli	0.00732919	0	0.50183229
Pipistrellus_nanulus	0.00731925	0	0.5018298
Pteropus_voeltzkowi	0.0073141	0	0.50182852
Saccolaimus_peli	0.00731261	0	0.50182814
Myotis_chiloensis	0.00730631	0	0.50182657
Rhinolophus_robinsoni	0.0072971	0	0.50182427
Pipistrellus_deserti	0.00729509	0	0.50182377
Monophyllus_plethodon	0.00729222	0	0.50182305
Paranyctimene_raptor	0.00728528	0	0.50182131
Ardops_nichollsi	0.00728124	0	0.5018203
Nyctophilus_geoffroyi	0.00727713	0	0.50181927
Taphozous_mauritianus	0.00727658	0	0.50181914
Pteropus_seychellensis	0.00727578	0	0.50181894
Pipistrellus_rusticus	0.00726721	0	0.50181679
Scotoecus_hirundo	0.00726574	0	0.50181643
Miniopterus_minor	0.00726241	0	0.50181559
Scotoecus_albofuscus	0.00725876	0	0.50181468
Choeroniscus_godmani	0.00725523	0	0.5018138
Ametrida_centurio	0.00725434	0	0.50181358
Molossus_pretiosus	0.00724685	0	0.5018117
Myotis_levis	0.0072457	0	0.50181142
Pteropus_hypomelanus	0.00723858	0	0.50180964
Kerivoula_argentata	0.007237	0	0.50180924

Hipposideros_lamottei	0.00723628	0	0.50180906
Rhinolophus_borneensis	0.00722855	0	0.50180713
Platyrrhinus_brachycephalus	0.00722681	0	0.5018067
Sphaeronycteris_toxophyllum	0.00722527	0	0.50180631
Myotis_ruber	0.00722299	0	0.50180574
Rhinolophus_cognatus	0.00722185	0	0.50180545
Otomops_wroughtoni	0.00722174	0	0.50180543
Artibeus_fimbriatus	0.00721761	0	0.50180439
Lampronycteris_brachyotis	0.0072168	0	0.50180419
Nyctiellus_lepidus	0.00720589	0	0.50180147
Nycteris_intermedia	0.00720441	0	0.5018011
Scotophilus_nux	0.00719142	0	0.50179785
Rhinophylla_fischerae	0.00719034	0	0.50179758
Chaerephon_leucogaster	0.00718786	0	0.50179696
Artibeus_cinereus	0.00718426	0	0.50179606
Artibeus_glaucus	0.00718182	0	0.50179545
Nyctimene_rabori	0.00717914	0	0.50179478
Vespertilio_murinus	0.0071489	0	0.50178722
Rhinolophus_nereis	0.0071434	0	0.50178584
Hipposideros_vittatus	0.00714228	0	0.50178556
Nyctinomops_laticaudatus	0.00714047	0	0.50178511
Rousettus_spinalatus	0.0071334	0	0.50178334
Centronycteris_maximiliani	0.00712749	0	0.50178187
Miniopterus_natalensis	0.00712589	0	0.50178146
Murina_tubinaris	0.00711671	0	0.50177917
Craseonycteris_thonglongyai	0.00710914	0	0.50177728
Melonycteris_melanops	0.00710327	0	0.50177581
Artibeus_concolor	0.00709753	0	0.50177438
Nyctimene_albiventer	0.00709043	0	0.5017726
Molossus_coibensis	0.00707845	0	0.50176961
Artibeus_anderseni	0.00707764	0	0.5017694
Hipposideros_fuliginosus	0.00707678	0	0.50176919
Lavia_frons	0.00707427	0	0.50176856
Corynorhinus_townsendii	0.00704464	0	0.50176115
Myotis_rosseti	0.00701788	0	0.50175446
Glauconycteris_poensis	0.00699432	0	0.50174857
Myotis_auriculus	0.00699362	0	0.5017484
Musonycteris_harrisoni	0.00698277	0	0.50174568
Chalinolobus_tuberculatus	0.00697289	0	0.50174322
Eptesicus_andinus	0.00697271	0	0.50174317
Sturnira_oporaphilum	0.00696599	0	0.50174149
Hipposideros_macrobullatus	0.00696245	0	0.50174061

Anoura_caudifer	0.00694692	0	0.50173672
Rhinolophus_swinnyi	0.00693828	0	0.50173456
Myotis_austroriparius	0.00693352	0	0.50173337
Carollia_subrufa	0.00693291	0	0.50173322
Rhinolophus_maclaudi	0.00692803	0	0.501732
Hipposideros_lankadiva	0.00692419	0	0.50173104
Choeronycteris_mexicana	0.00691084	0	0.5017277
Nyctimene_major	0.00690723	0	0.5017268
Vespadelus_caurinus	0.00686781	0	0.50171695
Carollia_colombiana	0.0068453	0	0.50171132
Lasiurus_xanthinus	0.00680803	0	0.501702
Rhynchonycteris_naso	0.00680271	0	0.50170067
Epomophorus_minimus	0.00680093	0	0.50170023
Nyctophilus_arnhemensis	0.00676865	0	0.50169216
Histiotus_macrotus	0.00674809	0	0.50168702
Myotis_melanorhinus	0.00674741	0	0.50168685
Triaenops_furculus	0.0067376	0	0.50168439
Hipposideros_stenotis	0.00670117	0	0.50167529
Glauconycteris_beatrix	0.00668902	0	0.50167225
Hipposideros_beatus	0.00668633	0	0.50167158
Eumops_underwoodi	0.00667676	0	0.50166918
Rousettus_madagascariensis	0.00664068	0	0.50166017
Vampyressa_melissa	0.00662635	0	0.50165658
Phyllostomus_latifolius	0.00662184	0	0.50165545
Molossops_mattogrossensis	0.00662078	0	0.50165519
Scotozous_dormeri	0.00661921	0	0.5016548
Hipposideros_dinops	0.00660573	0	0.50165143
Nyctophilus_walkeri	0.00660566	0	0.50165141
Myotis_thysanodes	0.00659717	0	0.50164929
Artibeus_amplus	0.00659126	0	0.50164781
Nycteris_nana	0.00659042	0	0.5016476
Myotis_goudoti	0.00658493	0	0.50164623
Rousettus_lanosus	0.00657885	0	0.50164471
Histiotus_velatus	0.00657579	0	0.50164394
Myotis_yumanensis	0.00657304	0	0.50164325
Platyrrhinus_vittatus	0.00656746	0	0.50164186
Hypsugo_eisentrauti	0.00656305	0	0.50164076
Rhinolophus_hipposideros	0.00654708	0	0.50163677
Chaerephon_johorensis	0.00654686	0	0.50163671
Mops_sarasinorum	0.0065376	0	0.5016344
Rhinolophus_marshalli	0.00652732	0	0.50163182
Rhinolophus_convexus	0.00649844	0	0.5016246

Mesophylla_macconnelli	0.00649842	0	0.5016246
Rhinolophus_shameli	0.00649568	0	0.50162392
Hipposideros_inornatus	0.0064901	0	0.50162252
Rhinolophus_silvestris	0.00648621	0	0.50162155
Rhinolophus_sakejiensis	0.00648454	0	0.50162113
Kerivoula_phalaena	0.00646971	0	0.50161742
Mops_spurrelli	0.00646879	0	0.50161719
Pteralopex_anceps	0.00645493	0	0.50161373
Hypsugo_macrotis	0.00644506	0	0.50161126
Chalinolobus_gouldii	0.00643664	0	0.50160916
Rhinolophus_deckenii	0.0064365	0	0.50160912
Saccopteryx_canescens	0.00643639	0	0.50160909
Erophylla_sezekorni	0.00642611	0	0.50160652
Aselliscus_stoliczkanus	0.00642152	0	0.50160538
Rhinolophus_adami	0.00642111	0	0.50160527
Mops_petersoni	0.00641661	0	0.50160415
Neoromicia_brunneus	0.00641661	0	0.50160415
Acerodon_humilis	0.00640486	0	0.50160121
Myopterus_whitleyi	0.00640081	0	0.5016002
Chaerephon_tomensis	0.00639999	0	0.50159999
Rhinolophus_sedulus	0.00637323	0	0.5015933
Rhinolophus_siamensis	0.006373	0	0.50159324
Rhinolophus_ziama	0.006373	0	0.50159324
Taphozous_kapalgensis	0.00636654	0	0.50159163
Peropteryx_leucoptera	0.00636164	0	0.50159041
Haplonycteris_fischeri	0.00636121	0	0.5015903
Nycteris_major	0.00636021	0	0.50159005
Mosia_nigrescens	0.00635995	0	0.50158998
Rhinolophus_denti	0.00635643	0	0.5015891
Rhinolophus_guineensis	0.00635216	0	0.50158804
Rhinolophus_darlingi	0.00634012	0	0.50158503
Rhinolophus_hillorum	0.00633611	0	0.50158402
Myotis_emarginatus	0.00632897	0	0.50158224
Nycteris_aurita	0.00632489	0	0.50158122
Eptesicus_nasutus	0.00632326	0	0.50158081
Platyrrhinus_dorsalis	0.00631841	0	0.5015796
Murina_fusca	0.00630922	0	0.5015773
Myotis_hajastanicus	0.00630922	0	0.5015773
Nyctalus_furvus	0.00630922	0	0.5015773
Chaerephon_solomonis	0.00630079	0	0.50157519
Tadarida_lobata	0.0063005	0	0.50157512
Myotis_moluccarum	0.00629446	0	0.50157361

Pteralopex_atrata	0.00629363	0	0.5015734
Pteropus_mahaganus	0.00629363	0	0.5015734
Sturnira_bogotensis	0.00629048	0	0.50157261
Murina_hilgendorfi	0.00628863	0	0.50157215
Myotis_punicus	0.00628863	0	0.50157215
Melonycteris_woodfordi	0.00628749	0	0.50157187
Myotis_schaubi	0.00628401	0	0.501571
Hipposideros_abae	0.00627328	0	0.50156832
Eptesicus_chiriquinus	0.006272	0	0.50156799
Pteropus_speciosus	0.00627016	0	0.50156754
Eumops_trumbulli	0.006257	0	0.50156424
Glauconycteris_kenyacola	0.00625587	0	0.50156396
Rhinolophus_montanus	0.00625383	0	0.50156345
Kerivoula_pellucida	0.00625323	0	0.5015633
Pteralopex_acrodonta	0.00625048	0	0.50156261
Micronycteris_brosseti	0.00624432	0	0.50156108
Pteropus_yapensis	0.00624396	0	0.50156099
Neoromicia_nanus	0.00624256	0	0.50156063
Anoura_geoffroyi	0.006238	0	0.5015595
Myotis_csorbai	0.00622281	0	0.5015557
Arielulus_cuprosus	0.00621565	0	0.50155391
Neoromicia_rendalli	0.00620488	0	0.50155122
Hipposideros_cervinus	0.00618993	0	0.50154748
Pteropus_neohibernicus	0.00618555	0	0.50154638
Myonycteris_brachycephala	0.00617607	0	0.50154401
Nycticeius_humeralis	0.00616959	0	0.50154239
Myotis_daubentonii	0.00616921	0	0.5015423
Nycteris_tragata	0.00615396	0	0.50153849
Cheiromeles_torquatus	0.00613231	0	0.50153307
Ptenochirus_minor	0.00613195	0	0.50153298
Artibeus_inopinatus	0.00610681	0	0.5015267
Vespadelus_douglasorum	0.00610642	0	0.5015266
Hipposideros_coronatus	0.00609962	0	0.5015249
Myonycteris_relicta	0.00609467	0	0.50152366
Hipposideros_durgadasi	0.00607333	0	0.50151833
Anthops_ornatus	0.00607308	0	0.50151827
Eptesicus_floweri	0.00607187	0	0.50151796
Acerodon_celebensis	0.00605757	0	0.50151439
Dobsonia_exoleta	0.00605757	0	0.50151439
Tadarida_australis	0.00602989	0	0.50150747
Pteropus_lombocensis	0.00602453	0	0.50150613
Mormoops_blainvillei	0.00602397	0	0.50150599

Nyctophilus_gouldi	0.00601622	0	0.50150405
Molossus_aztecus	0.00600514	0	0.50150128
Hipposideros_megalotis	0.00600284	0	0.50150071
Chilonatalus_micropus	0.00599833	0	0.50149958
Pteronotus_macleayii	0.00599833	0	0.50149958
Myotis_annectans	0.00599047	0	0.50149761
Glossophaga_morenoi	0.00599026	0	0.50149756
Pipistrellus_stenopterus	0.00596897	0	0.50149224
Chaerephon_ansorgei	0.00596838	0	0.50149209
Peropteryx_trinitatis	0.00596446	0	0.50149111
Hipposideros_sumbae	0.00596355	0	0.50149088
Pteropus_pilosus	0.00596307	0	0.50149076
Pteropus_subniger	0.00596307	0	0.50149076
Alionycteris_paucidentata	0.00596104	0	0.50149026
Pteropus_cognatus	0.00595163	0	0.5014879
Cheiromeles_parvidens	0.00594876	0	0.50148719
Hipposideros_curtus	0.00594837	0	0.50148709
Phyllonycteris_poeyi	0.00594463	0	0.50148615
Myotis_scotti	0.00594197	0	0.50148549
Rhogeessa_alleni	0.00594197	0	0.50148549
Triaenops_auritus	0.00593875	0	0.50148468
Taphozous_georgianus	0.00593702	0	0.50148425
Hipposideros_edwardshilli	0.00593408	0	0.50148352
Mormopterus_doriae	0.0059331	0	0.50148327
Lasiurus_pfeifferi	0.00592899	0	0.50148224
Nycticeius_cubanus	0.00592899	0	0.50148224
Lasiurus_insularis	0.00592899	0	0.50148224
Kerivoula_minuta	0.00592554	0	0.50148138
Phoniscus_atrox	0.00592554	0	0.50148138
Pteronotus_quadridens	0.00592165	0	0.50148041
Macrotus_waterhousii	0.00591955	0	0.50147988
Kerivoula_whiteheadi	0.00591741	0	0.50147935
Eptesicus_nilssonii	0.00591604	0	0.50147901
Mormopterus_minutus	0.00591338	0	0.50147834
Phyllops_falcatus	0.00591338	0	0.50147834
Nyctophilus_bifax	0.0059119	0	0.50147797
Myotis_yesoensis	0.00591023	0	0.50147755
Kerivoula_flora	0.0059062	0	0.50147655
Murina_suilla	0.0059062	0	0.50147655
Sturnira_mistratensis	0.0059036	0	0.50147589
Chironax_melanocephalus	0.0059035	0	0.50147587
Hypsugo_bodenheimeri	0.00590144	0	0.50147536

Hipposideros_thomensis	0.00588864	0	0.50147216
Chaerephon_bivittatus	0.00587981	0	0.50146995
Saccopteryx_bilineata	0.00587381	0	0.50146845
Myotis_abei	0.00587204	0	0.50146801
Neoromicia_tenuipinnis	0.00586778	0	0.50146694
Cynopterus_luzoniensis	0.0058645	0	0.50146612
Scotophilus_celebensis	0.0058645	0	0.50146612
Pteropus_griseus	0.0058645	0	0.50146612
Pipistrellus_adamsi	0.00586322	0	0.5014658
Pipistrellus_westralis	0.00586322	0	0.5014658
Pipistrellus_aero	0.00585816	0	0.50146454
Mormopterus_acetabulosus	0.00585816	0	0.50146454
Cynopterus_nusatenggara	0.00584995	0	0.50146248
Cloeotis_percivali	0.00584051	0	0.50146012
Euderma_maculatum	0.00583579	0	0.50145894
Pipistrellus_minahassae	0.00583455	0	0.50145863
Murina_rozendaali	0.00582997	0	0.50145749
Myotis_hermani	0.00582892	0	0.50145723
Otomops_johnstonei	0.00582562	0	0.5014564
Pteralopex_taki	0.00582562	0	0.5014564
Pteropus_aruensis	0.00582562	0	0.5014564
Pteropus_keyensis	0.00582562	0	0.5014564
Nyctimene_keasti	0.00582562	0	0.5014564
Myotis_dominicensis	0.00582461	0	0.50145615
Hypsugo_ariel	0.00581743	0	0.50145435
Epomophorus_minor	0.00581699	0	0.50145424
Pteropus_brunneus	0.00581233	0	0.50145308
Pteropus_tokudae	0.00581233	0	0.50145308
Pteropus_pohlei	0.00580042	0	0.5014501
Nyctimene_aello	0.00578744	0	0.50144686
Nyctimene_cephalotes	0.00578744	0	0.50144686
Nyctimene_vizcaccia	0.00578595	0	0.50144648
Pteropus_gilliardorum	0.00578595	0	0.50144648
Neoromicia_helios	0.00578123	0	0.5014453
Rhinopoma_muscatellum	0.00577744	0	0.50144436
Nyctophilus_heran	0.00577647	0	0.50144411
Micronycteris_minuta	0.00576664	0	0.50144166
Neoromicia_guineensis	0.0057549	0	0.50143872
Mops_trevori	0.00575077	0	0.50143769
Lasiurus_castaneus	0.00575014	0	0.50143753
Taphozous_hildegardeae	0.00574924	0	0.50143731
Dobsonia_chapmani	0.00572797	0	0.50143199

Chaerephon_aloysiisabaudiae	0.00572544	0	0.50143136
Chaerephon_jobensis	0.00572235	0	0.50143059
Mops_niangarae	0.00569134	0	0.50142283
Hipposideros_inexpectatus	0.00568073	0	0.50142018
Mops_niveiventer	0.00567805	0	0.50141951
Chaerephon_bemmeleni	0.00567203	0	0.50141801
Neoromicia_zuluensis	0.00566242	0	0.5014156
Mops_leucostigma	0.00565197	0	0.50141299
Myotis_nesopolus	0.00565122	0	0.5014128
Hypsugo_arabicus	0.00564991	0	0.50141247
Myotis_vivesi	0.00562538	0	0.50140634
Neoromicia_somalicus	0.00562054	0	0.50140513
Vampyressa_bidens	0.00561326	0	0.50140331
Chaerephon_nigeriae	0.00560387	0	0.50140097
Neoromicia_melckorum	0.00559569	0	0.50139892
Scotoecus_hindei	0.00559404	0	0.50139851
Scotophilus_robustus	0.00558753	0	0.50139688
Hipposideros_camerunensis	0.00558199	0	0.50139549
Neoromicia_flavescens	0.00558069	0	0.50139517
Scotoecus_albigula	0.00558069	0	0.50139517
Promops_nasutus	0.00558052	0	0.50139513
Otopteropus_cartilagonodus	0.00557931	0	0.50139483
Pteropus_intermedius	0.00557598	0	0.50139399
Hypsugo_crassulus	0.0055738	0	0.50139345
Eptesicus_bottae	0.00557217	0	0.50139304
Myotis_fortidens	0.00556974	0	0.50139243
Rhogeessa_parvula	0.00556974	0	0.50139243
Rhinopoma_macinnesi	0.00556965	0	0.50139241
Chaerephon_shortridgei	0.00555631	0	0.50138908
Hypsugo_anchietae	0.00555631	0	0.50138908
Chaerephon_russatus	0.00555328	0	0.50138832
Mops_brachypterus	0.00555325	0	0.50138831
Scotorepens_balstoni	0.005549	0	0.50138725
Hypsugo_lophurus	0.00554772	0	0.50138693
Artibeus_hirsutus	0.0055466	0	0.50138665
Acerodon_leucotis	0.00554018	0	0.50138504
Mops_demonstrator	0.00554014	0	0.50138503
Eptesicus_bobrinskoi	0.00554009	0	0.50138502
Hypsugo_anthonyi	0.00553695	0	0.50138423
Hypsugo_joffrei	0.00553695	0	0.50138423
Miniopterus_africanus	0.00553082	0	0.5013827
Mystacina_tuberculata	0.00552864	0	0.50138216

Eumops_bonariensis	0.00552643	0	0.5013816
Nyctimene_robinsoni	0.00552623	0	0.50138155
Hypsugo_vordermanni	0.00551539	0	0.50137884
Taphozous_troughtoni	0.00551531	0	0.50137882
Saccolaimus_mixtus	0.00551528	0	0.50137882
Eptesicus_gobiensis	0.00550973	0	0.50137743
Micronycteris_homezi	0.00550419	0	0.50137605
Epomophorus_angolensis	0.00550211	0	0.50137552
Chaerephon_bregullae	0.0055017	0	0.50137542
Hypsugo_kitcheneri	0.00550101	0	0.50137525
Dyacopterus_brooksi	0.00549356	0	0.50137339
Neopteryx_frosti	0.00549353	0	0.50137338
Dobsonia_pannietensis	0.00548992	0	0.50137248
Chaerephon_gallagheri	0.00548985	0	0.50137246
Murina_ussuriensis	0.0054877	0	0.50137192
Chaerephon_chapini	0.00547677	0	0.50136919
Hypsugo_musciculus	0.00547671	0	0.50136917
Otomops_secundus	0.00547559	0	0.50136889
Natalus_stramineus	0.00547258	0	0.50136814
Murina_florium	0.00546662	0	0.50136665
Pteropus_faunulus	0.00546598	0	0.50136649
Micropteropus_intermedius	0.00546251	0	0.50136563
Pteropus_molossinus	0.00545525	0	0.50136381
Pteropus_pelewensis	0.00544236	0	0.50136059
Sturnira_tildae	0.0054369	0	0.50135922
Diclidurus_albus	0.00543364	0	0.50135841
Mops_congicus	0.00543337	0	0.50135834
Bauerus_dubiaquercus	0.00542666	0	0.50135666
Pteropus_insularis	0.00542523	0	0.5013563
Hipposideros_maggietaylorae	0.00542362	0	0.5013559
Pteropus_banakrisi	0.00542154	0	0.50135538
Hipposideros_corynophyllus	0.00541851	0	0.50135463
Hipposideros_papua	0.00541551	0	0.50135387
Nyctimene_sanctacrucis	0.00541104	0	0.50135276
Pteropus_tuberculatus	0.00541104	0	0.50135276
Vampyressa_pusilla	0.00540111	0	0.50135027
Pteropus_mariannus	0.00539154	0	0.50134788
Chalinolobus_dwyeri	0.00539048	0	0.50134762
Nycteris_madagascariensis	0.0053848	0	0.5013462
Falsistrellus_tasmaniensis	0.00537066	0	0.50134266
Rousettus_bidens	0.00537019	0	0.50134255
Artibeus_fraterculus	0.0053568	0	0.5013392

Hipposideros_coxi	0.00535626	0	0.50133906
Nycteris_arge	0.00535535	0	0.50133883
Aselliscus_tricuspidatus	0.0053508	0	0.5013377
Hipposideros_doriae	0.0053508	0	0.5013377
Lasiurus_seminolus	0.00534834	0	0.50133708
Hipposideros_crumeniferus	0.00534297	0	0.50133574
Hipposideros_calcaratus	0.00533877	0	0.50133469
Diclidurus_ingens	0.00533851	0	0.50133462
Megaerops_wetmorei	0.00533797	0	0.50133449
Anoura_cultrata	0.00533635	0	0.50133408
Hipposideros_scutinares	0.00533571	0	0.50133393
Diclidurus_scutatus	0.00533348	0	0.50133337
Acerodon_mackloti	0.00533321	0	0.5013333
Epomophorus_grandis	0.0053327	0	0.50133317
Coelops_robinsoni	0.0053248	0	0.5013312
Taphozous_hilli	0.00532397	0	0.50133099
Hipposideros_grandis	0.00532095	0	0.50133024
Eptesicus_hottentotus	0.00531896	0	0.50132974
Saccopteryx_antioquensis	0.00531564	0	0.50132891
Dobsonia_anderseni	0.00531389	0	0.50132847
Dyacopterus_spadiceus	0.00531271	0	0.50132817
Promops_centralis	0.00531163	0	0.50132791
Diclidurus_isabellus	0.005309	0	0.50132725
Lonchorhina_marinkellei	0.00530758	0	0.50132689
Styloctenium_wallacei	0.00530714	0	0.50132678
Thoopterus_nigrescens	0.00530714	0	0.50132678
Harpyionycteris_celebensis	0.00530714	0	0.50132678
Rhogeessa_tumida	0.00530656	0	0.50132664
Hipposideros_rotalis	0.00530357	0	0.50132589
Pteropus_ualanus	0.00530282	0	0.5013257
Asellia_patrizii	0.00530229	0	0.50132557
Epomops_dobsonii	0.00529954	0	0.50132488
Aethalops_aequalis	0.005297	0	0.50132425
Arielulus_societatis	0.005297	0	0.50132425
Dobsonia_viridis	0.00529653	0	0.50132413
Penthetor_lucasi	0.00529635	0	0.50132408
Scoteanax_rueppellii	0.00529455	0	0.50132363
Hipposideros_semoni	0.00529361	0	0.5013234
Nyctimene_cyclotis	0.00529154	0	0.50132288
Platyrrhinus_aurarius	0.00529154	0	0.50132288
Nyctimene_certans	0.0052898	0	0.50132245
Nyctimene_draconilla	0.00528464	0	0.50132116

Hipposideros_dyacorum	0.0052817	0	0.50132042
Mormopterus_beccarii	0.00527902	0	0.50131975
Notopteris_macdonaldi	0.00527817	0	0.50131954
Nyctimene_masalai	0.00527817	0	0.50131954
Pteropus_anetianus	0.00527817	0	0.50131954
Vespadelus_darlingtoni	0.00527424	0	0.50131856
Rousettus_celebensis	0.00527303	0	0.50131826
Eptesicus_kobayashii	0.00527262	0	0.50131815
Myopterus_daubentonii	0.00527235	0	0.50131808
Nyctimene_malaitensis	0.00527146	0	0.50131786
Pteropus_ocularis	0.00527146	0	0.50131786
Pteropus_vetulus	0.00527146	0	0.50131786
Pteropus_woodfordi	0.00527146	0	0.50131786
Pteropus_melanopogon	0.00527146	0	0.50131786
Plecotus_auritus	0.00526369	0	0.50131592
Nycteris_vinsoni	0.00526235	0	0.50131558
Plerotes_anchietae	0.00526235	0	0.50131558
Syconycteris_carolinae	0.00526074	0	0.50131518
Emballonura_atrata	0.00525938	0	0.50131484
Chalinolobus_morio	0.00525674	0	0.50131418
Myotis_evotis	0.00525574	0	0.50131393
Dobsonia_crenulata	0.00525424	0	0.50131356
Dobsonia_emersa	0.00525424	0	0.50131356
Pteropus_caniceps	0.00525424	0	0.50131356
Pteropus_chrysoproctus	0.00525424	0	0.50131356
Pteropus_personatus	0.00525424	0	0.50131356
Nyctimene_minutus	0.00525417	0	0.50131354
Nyctinomops_femorosaccus	0.00525268	0	0.50131317
Kerivoula_intermedia	0.00525195	0	0.50131298
Lonchorhina_fernandezi	0.00525195	0	0.50131298
Hipposideros_marisae	0.00525153	0	0.50131288
Hipposideros_wollastoni	0.00524934	0	0.50131233
Pteropus_macrotis	0.00524613	0	0.50131153
Myotis_davidii	0.00524467	0	0.50131117
Histiotus_humboldti	0.00524467	0	0.50131117
Kerivoula_lenis	0.00524467	0	0.50131117
Lasiurus_salinae	0.00524467	0	0.50131117
Lasiurus_varius	0.00524467	0	0.50131117
Myotis_bucharensis	0.00524467	0	0.50131117
Myotis_nipalensis	0.00524467	0	0.50131117
Nycticeius_aenobarbus	0.00524467	0	0.50131117
Nyctophilus_howensis	0.00524467	0	0.50131117

Paranyctimene_tenax	0.00524467	0	0.50131117
Phyllonycteris_major	0.00524467	0	0.50131117
Plecotus_alpinus	0.00524467	0	0.50131117
Plecotus_kolombatovici	0.00524467	0	0.50131117
Plecotus_sardus	0.00524467	0	0.50131117
Rousettus_linduensis	0.00524467	0	0.50131117
Scotophilus_collinus	0.00524467	0	0.50131117
Lasiurus_atratus	0.00524467	0	0.50131117
Miniopterus_shortridgei	0.00524467	0	0.50131117
Mormoops_magna	0.00524467	0	0.50131117
Pteronotus_pristinus	0.00524467	0	0.50131117
Pteropus_loochoensis	0.00524467	0	0.50131117
Eptesicus_pachyotis	0.00524386	0	0.50131096
Eptesicus_matroka	0.00524198	0	0.50131049
Hesperoptenus_tomesi	0.00523701	0	0.50130925
Otomops_papuensis	0.00523701	0	0.50130925
Eumops_maurus	0.00523701	0	0.50130925
Kerivoula_africana	0.00523285	0	0.50130821
Miniopterus_paululus	0.00523285	0	0.50130821
Myotis_australis	0.00523285	0	0.50130821
Phoniscus_aerosa	0.00523285	0	0.50130821
Pipistrellus_sturdeei	0.00523285	0	0.50130821
Myotis_elegans	0.00523135	0	0.50130784
Syconycteris_hobbit	0.00522919	0	0.5013073
Chilonatalus_tumidifrons	0.00522689	0	0.50130672
Hipposideros_pelingensis	0.0052247	0	0.50130617
Hipposideros_demissus	0.0052247	0	0.50130617
Sturnira_mordax	0.00522282	0	0.5013057
Histiotus_alienus	0.00521984	0	0.50130496
Plecotus_balensis	0.00521953	0	0.50130488
Hipposideros_breviceps	0.00521941	0	0.50130485
Rhogeessa_mira	0.00521828	0	0.50130457
Vampyressa_nymphaea	0.00521756	0	0.50130439
Cynomops_mexicanus	0.00521497	0	0.50130374
Lonchophylla_mordax	0.00521367	0	0.50130342
Mormopterus_jugularis	0.00521087	0	0.50130271
Chalinolobus_nigrogriseus	0.00521053	0	0.50130263
Hipposideros_muscinus	0.00520684	0	0.50130171
Molossops_aequatorianus	0.00520609	0	0.50130152
Eudiscopus_denticulus	0.00520578	0	0.50130144
Kerivoula_eriophora	0.00520578	0	0.50130144
Myotis_morrisi	0.00520578	0	0.50130144

Sauromys_petrophilus	0.0052042	0	0.50130105
Arielulus_aureocollaris	0.00520248	0	0.50130062
Pteropus_fundatus	0.00520132	0	0.50130033
Pteropus_nitendiensis	0.00520132	0	0.50130033
Myotis_grisescens	0.00520048	0	0.50130012
Scotorepens_greyii	0.00519809	0	0.50129952
Laephotis_botswanae	0.00519809	0	0.50129952
Myotis_annamiticus	0.0051944	0	0.5012986
Natalus_primus	0.0051944	0	0.5012986
Anoura_luismanueli	0.0051944	0	0.5012986
Platalina_genovensium	0.00519387	0	0.50129846
Rhogeessa_gracilis	0.00518884	0	0.50129721
Myotis_planiceps	0.00518878	0	0.50129719
Pteropus_aldabrensis	0.00518867	0	0.50129717
Scotorepens_sanborni	0.00518661	0	0.50129665
Miniopterus_majori	0.00518102	0	0.50129525
Saccopteryx_gymnura	0.00518102	0	0.50129525
Mormopterus_loriae	0.00518081	0	0.5012952
Myotis_cobanensis	0.00518071	0	0.50129518
Rhogeessa_genowaysi	0.00518071	0	0.50129518
Myotis_ridleyi	0.00517442	0	0.5012936
Rhogeessa_minutilla	0.00517442	0	0.5012936
Emballonura_beccarii	0.00517442	0	0.5012936
Emballonura_raffrayana	0.00517442	0	0.5012936
Kerivoula_muscina	0.00517442	0	0.5012936
Mormopterus_norfolkensis	0.00517423	0	0.50129355
Nycteris_woodi	0.00517423	0	0.50129355
Phoniscus_papuensis	0.00517423	0	0.50129355
Eumops_patagonicus	0.00516959	0	0.50129239
Vespadelus_finlaysoni	0.00516959	0	0.50129239
Glauconycteris_humeralis	0.00516863	0	0.50129216
Pipistrellus_angulatus	0.00516863	0	0.50129216
Scotorepens_orion	0.00516844	0	0.50129211
Platymops_setiger	0.00516834	0	0.50129208
Myotis_stalkeri	0.00516787	0	0.50129197
Myotis_peninsularis	0.00516785	0	0.50129196
Molossus_barnesi	0.00516761	0	0.5012919
Rhogeessa_aeneus	0.00516503	0	0.50129126
Falsistrellus_mackenziei	0.00516199	0	0.5012905
Myotis_frater	0.0051617	0	0.50129042
Platyrrhinus_umbratus	0.00516078	0	0.50129019
Scleronycteris_ega	0.00516078	0	0.50129019

Vampyressa_brocki	0.00516078	0	0.50129019
Lasiurus_ebenus	0.00515988	0	0.50128997
Tadarida_kuboriensis	0.00515906	0	0.50128976
Nycteris_parisii	0.00515821	0	0.50128955
Otomops_madagascariensis	0.00515821	0	0.50128955
Taphozous_australis	0.00515821	0	0.50128955
Platyrrhinus_recifinus	0.00515597	0	0.50128899
Vespadelus_baverstocki	0.00515597	0	0.50128899
Mops_nanulus	0.00515524	0	0.50128881
Lophostoma_schulzi	0.00515515	0	0.50128879
Miniopterus_macrocneme	0.00514928	0	0.50128732
Murina_aenea	0.00514928	0	0.50128732
Nyctophilus_microtis	0.00514928	0	0.50128732
Balantiopteryx_infusca	0.00514912	0	0.50128728
Lonchophylla_bokermanni	0.00514894	0	0.50128723
Glauconycteris_machadoi	0.00514586	0	0.50128646
Histiotus_laephotis	0.00514586	0	0.50128646
Histiotus_magellanicus	0.00514586	0	0.50128646
Mimon_koepckeae	0.00514586	0	0.50128646
Myotis_atacamensis	0.00514586	0	0.50128646
Vespadelus_troughtoni	0.00514586	0	0.50128646
Myotis_macropus	0.00514586	0	0.50128646
Scotophilus_nucella	0.00514469	0	0.50128617
Pipistrellus_collinus	0.00514352	0	0.50128588
Pteralopex_pulchra	0.00514118	0	0.50128529
Miniopterus_gleni	0.00513786	0	0.50128446
Lonchorhina_orinocensis	0.00513701	0	0.50128425
Glauconycteris_gleni	0.00513698	0	0.50128424
Chalinolobus_picatus	0.0051323	0	0.50128307
Cistugo_lesueuri	0.0051323	0	0.50128307
Cistugo_seabrae	0.0051323	0	0.50128307
Eptesicus_innoxius	0.0051323	0	0.50128307
Laephotis_angolensis	0.0051323	0	0.50128307
Laephotis_namibensis	0.0051323	0	0.50128307
Mormopterus_kalinowskii	0.0051323	0	0.50128307
Mormopterus_phrudus	0.0051323	0	0.50128307
Myotis_aelleni	0.0051323	0	0.50128307
Tomopeas_ravus	0.0051323	0	0.50128307
Lonchophylla_dekeyseri	0.00513212	0	0.50128303
Lonchophylla_hesperia	0.00513212	0	0.50128303
Sturnira_nana	0.00513212	0	0.50128303
Lonchophylla_handleyi	0.00513212	0	0.50128303

Emballonura_furax	0.00512523	0	0.50128131
Emballonura_serii	0.00512511	0	0.50128127
Melonycteris_fardoulisi	0.00512511	0	0.50128127
Nyctophilus_nebulosus	0.00512511	0	0.50128127
Pteropus_temminckii	0.00512511	0	0.50128127
Taphozous_achates	0.00512511	0	0.50128127
Chalinolobus_neocaledonicus	0.00512511	0	0.50128127
Glauconycteris_superba	0.00512433	0	0.50128108
Hesperoptenus_gaskelli	0.00512433	0	0.50128108
Falsistrellus_petersi	0.00512236	0	0.50128059
Myotis_gomantongensis	0.00512108	0	0.50128027
Pipistrellus_papuanus	0.00512108	0	0.50128027
Pipistrellus_wattsi	0.00512108	0	0.50128027
Platyrrhinus_chocoensis	0.00512108	0	0.50128027
Rhogeessa_hussoni	0.00512108	0	0.50128027
Lonchorhina_inusitata	0.00512108	0	0.50128027
Pipistrellus_inexspectatus	0.00511891	0	0.50127973
Molossus_sinaloae	0.00511729	0	0.50127932
Kerivoula_agnella	0.0051116	0	0.5012779
Kerivoula_myrella	0.0051116	0	0.5012779
Micronycteris_sanborni	0.00510888	0	0.50127722
Thyroptera_lavali	0.00510888	0	0.50127722
Glauconycteris_curryae	0.00510881	0	0.5012772
Micronycteris_matses	0.00510881	0	0.5012772
Emballonura_dianae	0.00510758	0	0.50127689
Hesperoptenus_doriae	0.00510758	0	0.50127689
Nyctophilus_microdon	0.00510758	0	0.50127689
Pharotis_imogene	0.00510758	0	0.50127689
Glauconycteris_alboguttata	0.00510758	0	0.50127689
Artibeus_incomitatus	0.00509965	0	0.50127491
Choeroniscus_periosus	0.00509535	0	0.50127384
Glauconycteris_egeria	0.00509535	0	0.50127384
Kerivoula_cuprosa	0.00509535	0	0.50127384
Neonycteris_pusilla	0.00509524	0	0.50127381
Glyphonycteris_behnii	0.00509518	0	0.50127379
Rhinophylla_alethina	0.00509518	0	0.50127379
Myotis_findleyi	0.00508622	0	0.50127155
Pteropus_rennelli	0.00506068	0	0.50126517
Rhinonicteris_aurantia	0.00505694	0	0.50126423
Miniopterus_robustior	0.00504735	0	0.50126183
Myotis_insularum	0.00504735	0	0.50126183
Lophostoma_evotis	0.00502997	0	0.50125749

Amorphochilus_schnablii	0.00502845	0	0.50125711
Rhinolophus_rouxii	0.00499813	0	0.50124953
Pteropus_capistratus	0.00492376	0	0.50123094
Aproteles_bulmerae	0.00492118	0	0.50123029
Vespadelus_vulturnus	0.00491543	0	0.50122886
Triaenops_rufus	0.00487991	0	0.50121998
Vespadelus_regulus	0.0048666	0	0.50121665
Lasiurus_intermedius	0.00486637	0	0.50121659
Epomophorus_crypturus	0.00484558	0	0.50121139
Pteropus_admiralitatum	0.00483223	0	0.50120806
Myzopoda_aurita	0.00482564	0	0.50120641
Nyctophilus_timoriensis	0.00482289	0	0.50120572
Notopteris_neocaledonica	0.00474365	0	0.50118591
Myotis_sodalis	0.00473159	0	0.5011829
Hipposideros_turpis	0.00472571	0	0.50118143
Neoromicia_capensis	0.00462729	0	0.50115682
Balantiopteryx_io	0.00442882	0	0.5011072
Mormopterus_planiceps	0.00441811	0	0.50110453
Vespadelus_pumilus	0.00438333	0	0.50109583
Dobsonia_beauforti	0.00435885	0	0.50108971
Vampyressa_thyone	0.00431884	0	0.50107971
Hylonycteris_underwoodi	0.00414261	0	0.50103565
Scotonycteris_ophiodon	0.00404804	0	0.50101201
Casinycteris_argynnis	0.00400937	0	0.50100234
Idionycteris_phyllotis	0.00397627	0	0.50099407
