# Supplementary material for: Undiscovered Bat Hosts of Filoviruses
Source: PLoS Negl Trop Dis. 2016 Jul 14;10(7):e0004815. doi: 10.1371/journal.pntd.0004815 (PMC4945033; doi:10.1371/journal.pntd.0004815)
Supplement: S4 Table — (PDF) [file pntd.0004815.s005.pdf]

%data cells dropped	S statistic	Rho	p-value
1	3045800	0.9868521	< 2.2e-16
5	2475800	0.9893126	< 2.2e-16
10	7437700	0.967893	< 2.2e-16
15	7329400	0.9683606	< 2.2e-16
20	22637000	0.9022795	< 2.2e-16
