# Supplementary material for: Undiscovered Bat Hosts of Filoviruses
Source: PLoS Negl Trop Dis. 2016 Jul 14;10(7):e0004815. doi: 10.1371/journal.pntd.0004815 (PMC4945033; doi:10.1371/journal.pntd.0004815)
Supplement: S5 Table — The final model includes the number of citations per bat species in Web of Science (WOS_HITS), showing that while study effort is within the top dozen most important predictors of filovirus-positive status, it is less important than intrinsic traits. Variables taken from PanTHERIA retain their original names. We also report here the baseline AUC and standard deviation calculated from a bootstrapped randomized permutation analysis. (PDF) [file pntd.0004815.s006.pdf]

**Response**	Label
**Error distribution**	Bernoulli
**Train/test fraction**	80/20
**Shrinkage**	0.001
**Interaction depth**	3
**Best.iter**	2356/5000
**CV folds**	10
**Performance (train)**	0.995
**Performance (test)**	0.974
**Mean(permutedAUC500)**	0.6 (sd=0.08)
**Corrected test AUC**	**0.874**

**Variable**	**Relative Importance**
spp.dens.per.km	13.11331244
X23.1_SexualMaturityAge_d	11.84181085
X5.3_NeonateBodyMass_g	9.59740908
X10.1_PopulationGrpSize	8.826269923
X5.4_WeaningBodyMass_g	8.134868537
Log10.mass_specific_production.	7.684370389
X27.2_HuPopDen_Mean_n.km2	4.401490125
X26.1_GR_Area_km2	4.035338776
X27.4_HuPopDen_Change	3.268129917
X26.5_GR_MaxLong_dd	2.613752557
WOS_HITS	2.537961838
X9.1_GestationLen_d	1.941994599
X3.1_AgeatFirstBirth_d	1.918571991
X16.1_LittersPerYear	1.701514746
X15.1_LitterSize	1.577198076
X5.1_AdultBodyMass_g	1.370461379
X26.3_GR_MinLat_dd	1.265506038
X26.2_GR_MaxLat_dd	1.232562947
X25.1_WeaningAge_d	1.092707981
X28.1_Precip_Mean_mm	1.082682164
X8.1_AdultForearmLen_mm	1.027033826
X26.4_GR_MidRangeLat_dd	0.956366489
X26.6_GR_MinLong_dd	0.947111109
aridity	0.841975573
X28.2_Temp_Mean_01degC	0.767619668
X30.2_PET_Mean_mm	0.611492891
X27.3_HuPopDen_5p_n.km2	0.551542133
BodySizeRatio	0.517049908
X13.1_AdultHeadBodyLen_mm	0.503954943
postnatGR	0.414734305
X6.2_TrophicLevel	0.412999422
X30.1_AET_Mean_mm	0.39804641
X26.7_GR_MidRangeLong_dd	0.355652208
Diet.Fruit	0.283731119
RelAgeSexMat	0.273870251
X13.2_NeonateHeadBodyLen_mm	0.265575067
X27.1_HuPopDen_Min_n.km2	0.252911806
Rhinolophidae	0.185631509
X6.1_DietBreadth	0.180218346
BodyMass.Value	0.177057682
Pteropodidae	0.14543347
X10.2_SocialGrpSize	0.118735711
X17.1_MaxLongevity_m	0.11464902
X2.1_AgeatEyeOpening_d	0.073629401
X18.1_BasalMetRate_mLO2hr	0.071359519
Vespertilionidae	0.06771355
migration status	0.058279096
Diet.Inv	0.048245038
ForStrat.Value	0.046545423
X5.5_AdultBodyMass_g_EXT	0.038010393
torpor	0.033928007
Molossidae	0.012454492
Hipposideridae	0.005112458
IUCN	0.003415404
X1.1_ActivityCycle	0
X5.2_BasalMetRateMass_g	0
Phyllostomidae	0
